# Cervicovaginal microbiota and metabolome predict preterm birth risk in an ethnically diverse cohort

**DOI:** 10.1172/jci.insight.149257

**Published:** 2021-08-23

**Authors:** Flavia Flaviani, Natasha L. Hezelgrave, Tokuwa Kanno, Erica M. Prosdocimi, Evonne Chin-Smith, Alexandra E. Ridout, Djuna K. von Maydell, Vikash Mistry, William G. Wade, Andrew H. Shennan, Konstantina Dimitrakopoulou, Paul T. Seed, A. James Mason, Rachel M. Tribe

**Affiliations:** 1 Department of Women and Children’s Health, School of Life Course Sciences, Faculty of Life Sciences and Medicine,; 2 Translational Bioinformatics Platform, NIHR Biomedical Research Centre, Guy’s and St. Thomas’ NHS Foundation Trust,; 3 Institute of Pharmaceutical Science, School of Cancer and Pharmaceutical Sciences, Faculty of Life Sciences and Medicine, and; 4 Centre for Host-Microbiome Interactions, Faculty of Dentistry, Oral & Craniofacial Sciences, King’s College London, London, United Kingdom.

**Keywords:** Microbiology, Reproductive Biology, Bioinformatics, Obstetrics/gynecology, Translation

## Abstract

The syndrome of spontaneous preterm birth (sPTB) presents a challenge to mechanistic understanding, effective risk stratification, and clinical management. Individual associations between sPTB, self-reported ethnic ancestry, vaginal microbiota, metabolome, and innate immune response are known but not fully understood, and knowledge has yet to impact clinical practice. Here, we used multi–data type integration and composite statistical models to gain insight into sPTB risk by exploring the cervicovaginal environment of an ethnically heterogenous pregnant population (*n =* 346 women; *n =* 60 sPTB < 37 weeks’ gestation, including *n =* 27 sPTB < 34 weeks). Analysis of cervicovaginal samples (10–15^+6^ weeks) identified potentially novel interactions between risk of sPTB and microbiota, metabolite, and maternal host defense molecules. Statistical modeling identified a composite of metabolites (leucine, tyrosine, aspartate, lactate, betaine, acetate, and Ca^2+^) associated with risk of sPTB < 37 weeks (AUC 0.752). A combination of glucose, aspartate, Ca^2+^, *Lactobacillus crispatus*, and *L*. *acidophilus* relative abundance identified risk of early sPTB < 34 weeks (AUC 0.758), improved by stratification by ethnicity (AUC 0.835). Increased relative abundance of *L*. *acidophilus* appeared protective against sPTB < 34 weeks. By using cervicovaginal fluid samples, we demonstrate the potential of multi–data type integration for developing composite models toward understanding the contribution of the vaginal environment to risk of sPTB.

## Introduction

The syndrome of spontaneous preterm birth (sPTB) is a major cause of neonatal death and morbidity globally (1). Considerable effort has been directed toward defining the causal mechanisms responsible for the different manifestations of this complex syndrome (2, 3), with inflammation and/or infection consistently emerging as important factors for midtrimester miscarriage, early sPTB (<34 weeks), and preterm prelabor rupture of membranes (PPROM; refs. 4–7). The contribution of the vaginal microbiota/microbiome to sPTB, although the focus of many studies (8–17), has yet to result in a clear understanding of the pathophysiology — or in the identification of effective biomarkers and clinical interventions to improve pregnancy outcomes (18).

Associations between vaginal bacterial communities, bacterial vaginosis (BV), and preterm birth are widely reported (12, 15, 16). Distinct vaginal bacterial communities have been identified in nonpregnant and pregnant women by means of 16S rRNA gene amplicon sequencing and community state type (CST) classifications (17, 19). Typically, a healthy vaginal ecosystem and term birth has been described as a *Lactobacillus*-dominated microbiome, with microbiota metabolites playing important roles in inhibition of bacterial and viral infections (14, 20) — e.g., *Lactobacillus* *crispatus* contributes to low pH by secreting the metabolite D-lactate (21). In contrast, vaginal dysbiosis (with or without symptoms of BV), by disrupting the ecological equilibrium, has been proposed to induce local inflammation and risk of invasion by infectious agents potentially increasing sPTB risk (22, 23). Low relative abundance of *L*. *crispatus*, with raised *L*. *iners* and acetate — together with low levels of succinate and lactate — have been implicated in this (15). However, BV does not explain all sPTB, conferring only a 2-fold increase in risk (24), and not all sPTB deliveries present a microbial profile dominated by *L*. *iners* (11). This is particularly apparent among Black women, where mixed bacterial vaginal communities are common even before pregnancy (9, 12, 25, 26), but a relative lack of lactobacilli does not appear to explain the higher rate of sPTB in a US study of Black women (10). Knowledge of the vaginal microbiota alone seems insufficient to develop sPTB prediction tools, and a much greater understanding of the vaginal microbiota and the environment within which it lives (i.e., maternal host response) is clearly needed.

Previously, in pregnant women with a history of sPTB or late miscarriage, we explored the contribution of the maternal host response to risk of preterm birth (27–30). Host defense peptides (HDPs), including trappin2/elafin (referred to here as elafin, a protein regulated by tryptases and inhibitor of human neutrophil elastase [HNE]) and cathelicidin (a peptide synthesized by epithelial cells and coreleased with HNE from neutrophils) were raised in cervicovaginal fluid (CVF; refs. 27, 28, 30). The stimuli for these innate host responses were not directly investigated, but we proposed a role for vaginal dysbiosis and suggested that inflammation could contribute to sPTB risk through cervical collagen remodeling. Bacterial metabolites could also compromise cervicovaginal defenses via modulation of host response and epithelial function (20).

In this study, we have explored interactions between the cervicovaginal metabolic environment and microbiota in tandem with the host innate immune response in a prospective United Kingdom (UK) longitudinal cohort of pregnant women (INSIGHT). A combination of single data type and integrative analyses was performed to understand the relation between specific components of the vaginal environment and risk of sPTB (<34 weeks [sPTB34]; <37 weeks [sPTB37]) both in the whole community and stratified by self-reported ethnic ancestry (based on UK national census groups; ref. 31), with the goal of supporting development of sPTB prediction tools and treatments that could be applied in clinical settings.

## Results

### Study participant demographics.

Demographic details of the pregnant participants and corresponding CVF samples are presented in Supplemental Table 1 (supplemental material available online with this article; https://doi.org/10.1172/jci.insight.149257DS1). CVF analysis provided matched bacterial (16S rRNA gene), metabolome (^1^H-NMR), and biochemical (pH, HDPs, and HNE) data sets at study entry (10–15^+6^ weeks gestation) and later in pregnancy (16–23^+6^ weeks). A total of 346 women provided at least 1 sample during early (10–15^+6^ weeks) and/or later (16-23^+6^ weeks) gestation for analysis (Supplemental Figure 1). The self-reported ethnicities for this UK-based cohort were: White (68.2%), Black (British, African, or Caribbean, 21.7%), and Other (Indian, Pakistani, Bangladeshi, Arab, Chinese, South East Asian, other unreported/unclassified, 10.1%). Most sPTB cases (<37 weeks, which included midtrimester miscarriage, *n =* 60) originated in the high-risk group (women recruited from preterm birth surveillance clinics) (Supplemental Table 1). Differences in BMI could be observed between different self-reported ethnicities with an increase in Black women, but this was irrespective of pregnancy outcome (Supplemental Figure 2).

### Relationship between CVF microbiota communities and metabolites.

Grouping of samples by principal coordinates analysis (PCoA) (Supplemental Figure 3) differed slightly from the CST identified and reported by Ravel et al. (17) as follows: PCoA group A (dominated by *L*. *crispatus*), group B (*L*. *gasseri*), group C (*L*. *iners*), group D (a range of diverse bacteria), and group E (predominance of both *L*. *crispatus* and *L*. *gasseri*) (Supplemental Figure 4). *L*. *jensenii* was found in high abundance in some of the samples belonging to PCoA groups C and D; it was present in 91% of the samples but reached 10% of the community in 13.6% of samples, while it dominated the community in only 0.57%.

**Figure 1:**
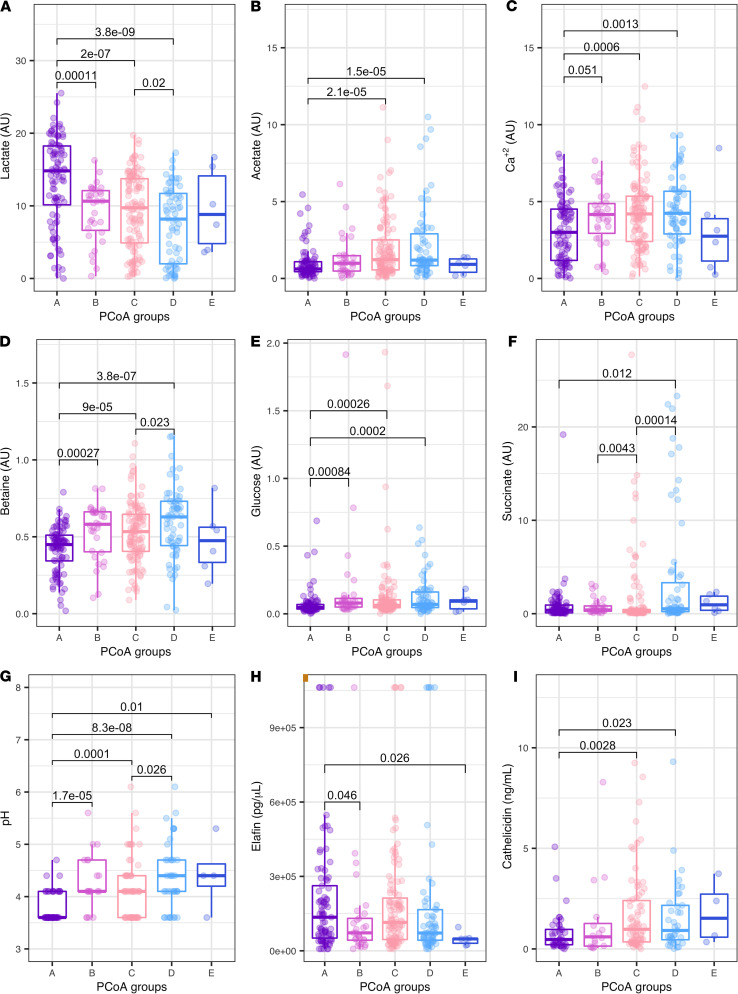
Whole cohort cervicovaginal fluid (CVF) components relationships. CVF metabolites, pH, and host defense peptides explored in relation with bacterial composition based on Principal Coordinates Analysis (PCoA) groups in early pregnancy (10–15^+6^ weeks). Wilcoxon comparison shown if *P* < 0.05. (**A**–**I**) lactate, acetate, Ca^2+^, betaine glucose, succinate, pH, elafin, and cathelicidin (high-risk women only). Number of samples (*n*) per PCoA group comparisons as follows: (**A**–**F**) PCoA A = 89, PCoA B = 31, PCoA C = 115, PCoA D = 64, and PCoA E = 6. (**G**) PCoA A = 55, PCoA B = 19, PCoA C = 62, PCoA D = 39, and PCoA E = 4. (**H**) PCoA A = 85, PCoA B = 29, PCoA C = 111, PCoA D = 61, and PCoA E = 6. (**I**) PCoA A = 41, PCoA B = 16, PCoA C = 76, PCoA D = 37, and PCoA E = 4. *Y* axis represents the normalized NMR peaks (arbitrary units, au) (**A**–**F**), pg/μL (**H**), and ng/mL (**I**). Horizontal line and boxes represent median and IQR.

Integration of metabolites with PCoA groups demonstrated strong relationships between individual metabolites and bacterial composition (significant comparisons shown in Supplemental Table 2). Women with a high prevalence of *L*. *crispatus* (PCoA A), as expected, presented significantly higher levels of lactate in comparison with women assigned to PCoA groups B or C; lactate levels were also increased in PCoA C compared with PCoA D (Figure 1A). The pH profile reflected the association with lactate (Figure 1, A and G). In contrast, metabolites such as acetate, Ca^2+^ (identified through its binding to EDTA), betaine, glucose, and succinate showed an opposing pattern with lower concentrations in women presenting dominance of *L*. *crispatus* (Figure 1, B–F). When stratified by ethnicity (Black and White women), Orthogonal Projections to Latent Structures — Discriminant Analysis (OPLS-DA) of the 28 metabolites from the CVF nuclear magnetic resonance (NMR) showed differences at both time points (Supplemental Table 3 and Supplemental Figure 5). Specifically, White women exhibited significantly lower Ca^2+^ (*P <* 0.05; Supplemental Figure 5, A and B) and higher lactate in later pregnancy (*P <* 0.05; Supplemental Figure 5C).

### Relationship between CVF microbiota communities, metabolites, and pregnancy outcome.

**Figure 2:**
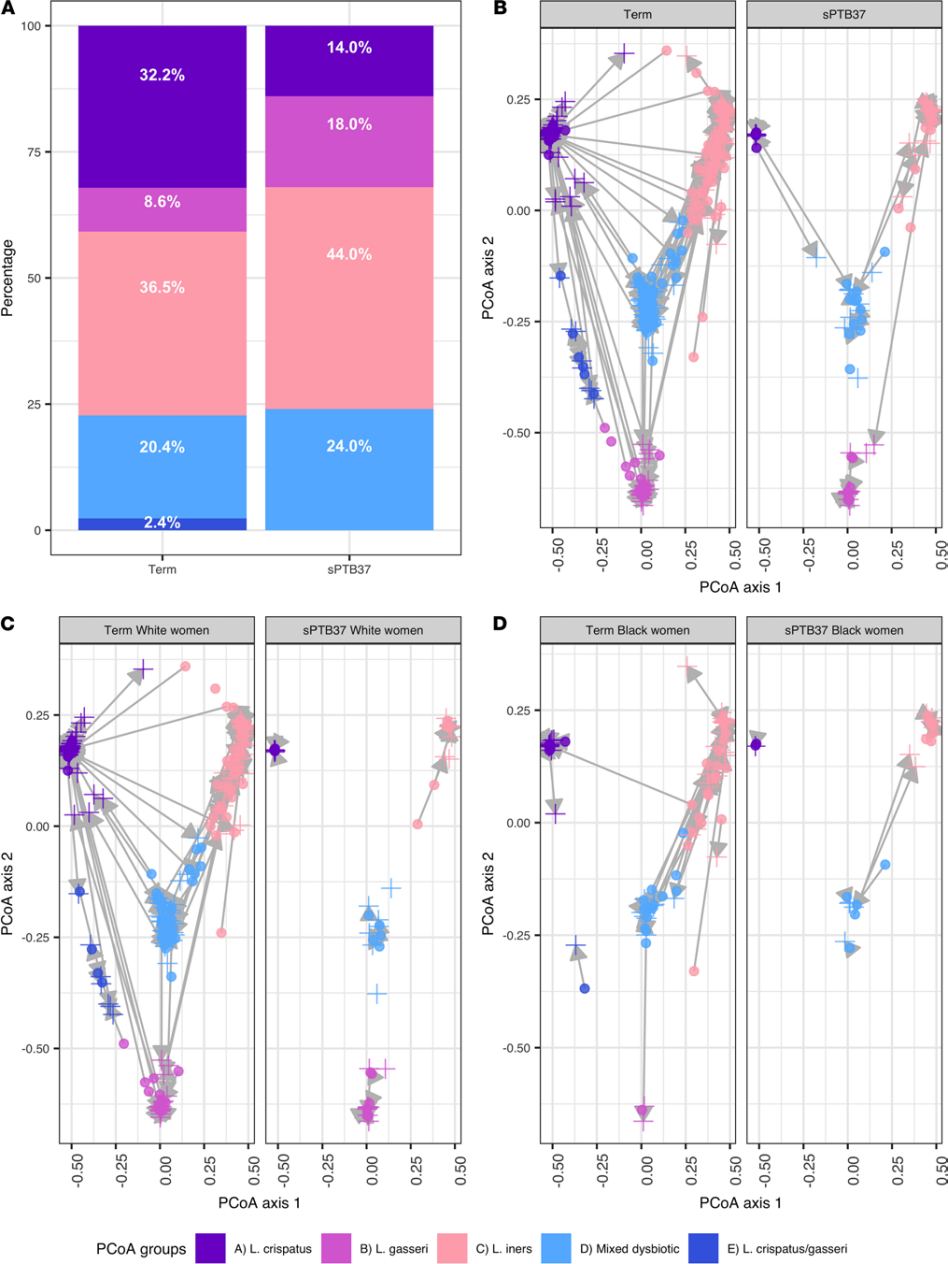
Bacterial composition of the cervicovaginal fluid based on Principal Coordinates Analysis (PCoA) groups and stratified by pregnancy outcome. (**A**) Distribution of PCoA groups in the whole community based on delivery outcome for term birth and spontaneous preterm birth before 37 weeks’ gestation (sPTB37) in early samples (10–15^+6^ weeks). (**B**–**D**) Dynamics of PCoA groups during pregnancy as identified in the early and late (16–23^+6^ weeks) sampling times in relation to delivery outcome for the whole community (**B**), for White women (**C**), and Black women (**D**). Circles represents early samples, and crosses represent late samples. (**A** and **B**) Early samples term, *n* = 255, and sPTB37, *n* = 50; late samples term, *n* = 263, and sPTB37, *n* = 50. (**C**) White women early samples term, *n* = 184, and sPTB37, *n* = 23; late samples term, *n* = 190, and sPTB37, *n* = 28. (**D**) Black women early samples term, *n* = 51, and sPTB37, *n* = 16; late samples term, *n* = 52, and sPTB37, *n* = 13.

sPTB37 was associated with a lower abundance of PCoA group A and an increase in group C at both sampling gestations (Figure 2, A and B, and Supplemental Figure 6). For high-risk women who later developed a short cervix during pregnancy (a known risk factor for sPTB), their baseline PCOA was compared with other high-risk women who did not develop a short cervix (Supplemental Figure 7). In early pregnancy, operational taxonomic unit (OTU) composition was significantly different for pregnancy outcome (term versus sPTB37) (Permutational multivariate analyses [PERMANOVA] early and late *P <* 0.005).

White women delivering < 37 weeks exhibited a shift from PCoA A to PCoA B (Figure 2C and Supplemental Figure 7). However, regardless of outcome, the CVF of Black women was mainly characterized by PCoA groups C or D during early and late gestations (Supplemental Figure 8). Both Black and White women delivering sPTB37 presented more stable PCoA groups (Figure 2, C and D), with reduced changes in bacterial community between early and late pregnancy. Differences in OTU composition detected in term and sPTB37 for White and Black cohorts are illustrated in Supplemental Figures 9 and 10.

Differences in OTU profiles were analyzed using the linear discriminant analysis (LDA) effect size (LEfSe). LEfSe analyses based on term outcome for the whole community allowed the identification of OTU_1 (consensus identification *L*. *crispatus*) as an indicator of term outcome in both early and late samples and OTU_6 (*L*. *acidophilus*) in earlier samples, while OTU_18 (*Prevotella bivia*) and OTU_27 (*L*. *delbrueckii)* were identified as associated with sPTB37 (Supplemental Table 4). When separating the data set based on racial or ethnic groups, OTU_1 (*L*. *crispatus*) alone was associated with term birth both in early and late samples in White women. However, for Black women, it was only possible to determine OTUs associated with term pregnancy in late samples, with several OTUs associated with preterm outcome in both visits (Supplemental Table 4). Low relative abundance of OTU_6 (*L*. *acidophilus*) was associated with sPTB37 (mean 0.004 ± 0.007). OTUs associated with sPTB34 weeks are shown in Supplemental Table 5.

OPLS-DA analyses did not show differences in the metabolic profile of women who delivered sPTB37 compared with term women. However, using the univariate Cox model, we identified features related to pregnancy outcome (Table 1). Analyses showed that acetate and Ca^2+^ are features able to identify and distinguish between term and sPTB37 with other metabolites, including with aspartate associated with sPTB34. Lactate was also identified — but only in late samples within the whole cohort; this most likely reflects the previously report gestational shift toward *Lactobacillus* spp. (8) and more specifically *L*. *crispatus* dominance (11).

### Relationship between CVF host response (HDPs), microbiota, and metabolome.

CVF measurements of the host response (elafin and HNE) at study entry (early pregnancy), originating from a subset of the data published for the INSIGHT cohort (30), were utilized to assess relationships with microbial community and metabolic composition. Elafin concentrations were significantly increased in women with PCoA group A compared with groups B and E (Figure 1H), suggesting elafin plus the presence of *L*. *crispatus* is protective and is potentially modulated by bacteria or metabolites present within other PCoA communities. Inverse patterns are observed for cathelicidin (measured in high-risk women only) where PCoA A presented lower concentrations in relation to groups C and D (Figure 1I). No significant relationship was observed for HNE with bacterial PCoA groups. CVF inflammation was higher in Black women compared with White women. Elafin concentrations in Black women were raised during both visits (*P*_Kruskal-Wallis_ < 0.05). Cathelicidin concentrations (only measured in high-risk women) were also significantly increased in late pregnancy in Black women compared with White women (*P*_Kruskal-Wallis_ < 0.01).

**Figure 3:**
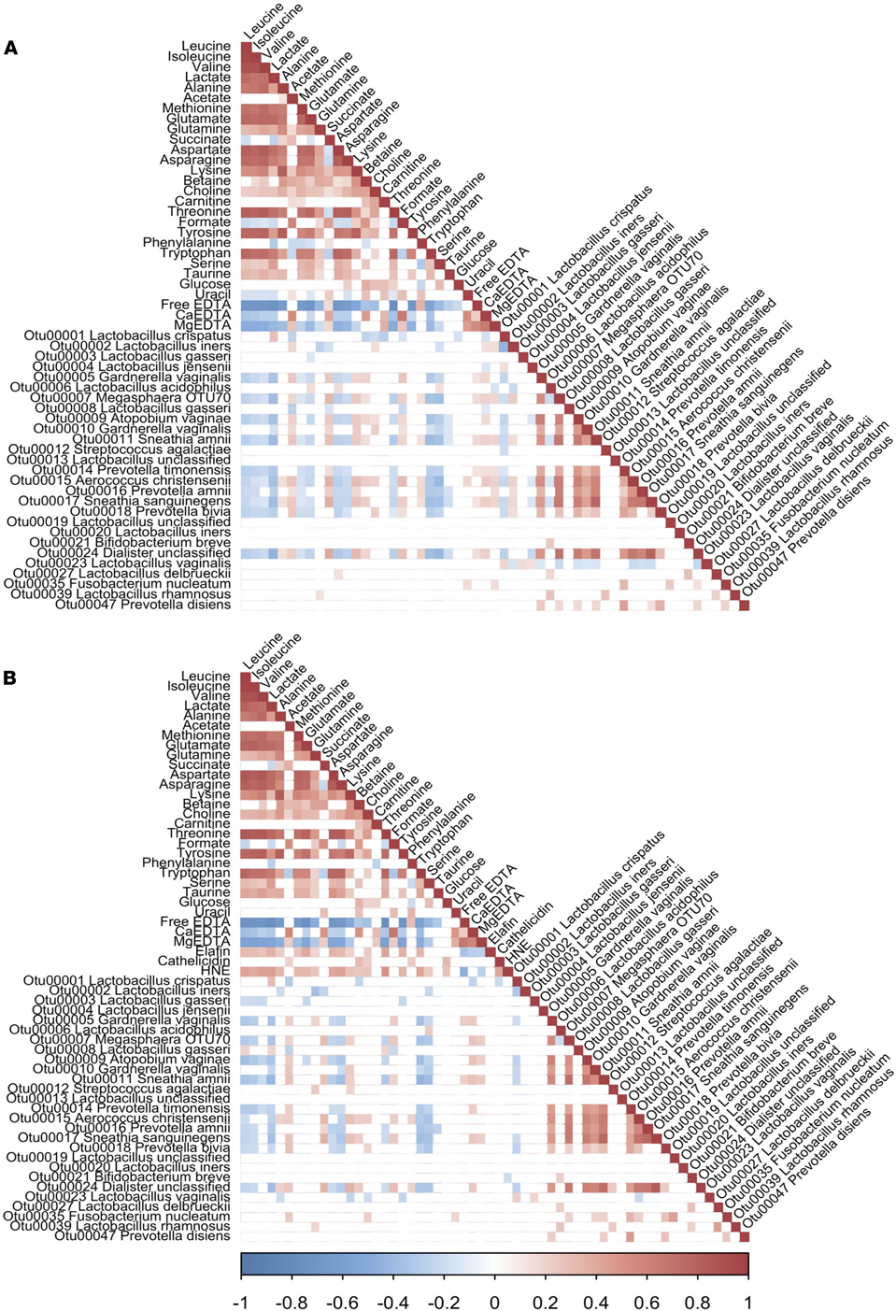
Spearman’s correlation analyses of early gestation cervicovaginal fluid. (**A** and **B**) OTUs and metabolites (*n* = 305) and OTUs, metabolites, elafin, cathelicidin, and HNE (*n =* 161). OTUs selected as follows: showing more than 1% average abundance; identified via LEfSe analyses as associated with spontaneous preterm birth (sPTB < 37 weeks). Only correlations with adjusted *P* < 0.05 are shown; scale represents correlations values: blue (negative) and red (positive).

CVF metabolites provide a summary readout of the functional impact of complex bacterial communities on the vaginal environment, and we interrogated this relationship through Spearman’s correlation analyses of vaginal OTUs (most abundant OTUs and some identified through LEfSe in comparison with sPTB37) and metabolites (Figure 3A and Supplemental Figure 11A). This was repeated using data from high-risk women only so that cathelicidin interactions could be assessed (Figure 3B and Supplemental Figure 11B). Subsequently, a similar analysis was run to include CVF pH data (Supplemental Figure 12, A and B). Several OTUs significantly correlated with individual metabolites after adjusting *P* values. Notably, OTU_6 (*L*. *acidophilus*) and OTU_27 (*L*. *delbrueckii*) were each positively associated with the aspartate signal (Figure 3A); OTU_6 (*L*. *acidophilus*), in turn, was negatively correlated with OTU_2 (*L*. *iners*). OTU_1 presented positive correlation with lactate and negative correlation with acetate, glucose, and OTU_2. OTU_2 and OTU_20 (*L*. *iners*) showed relatively few significant correlations with other OTUs and metabolites. Conversely, OTU_5 and OTU_10 (*Gardnerella vaginalis group*), OTU_7 (*Megasphaera* “OTU70”)*,* OTU_9 (*Atopobium vaginae*), OTU_11 (*Sneathia amnii*), OTU_15 (*Aerococcus christensenii*), OTU_16 (*Prevotella amnii*), OTU_17 (*Sneathia sanguinegens*) and OTU_24 (*Dialister* unclassified; Figure 3A) were negatively correlated with lactate and aspartate, while positively correlated with acetate, Ca^2+^ (Figure 3A), and pH (Supplemental Figure 12A). There were other, but differing, correlations for members of this OTU cluster with other metabolites (e.g., succinate and serine). OTU_18 (*Prevotella bivia*) showed some overlapping positive correlations with this above the OTU cluster — alongside choline, formate (Figure 3A), and pH (Supplemental Figure 12A) — and a significant positive correlation with acetate at both time points (Figure 3A and Supplemental Figure 11A).

Ca^2+^, which emerged as significant in a variety of our analyses (e.g., feature selection and univariate), correlated positively with acetate, succinate, betaine, choline, carnitine, formate and uracil and OTU_7 (*Megasphaera* “OTU70”), OTU_9 (*Atopobium vaginae*), OTU_11 (*Sneathia amnii*), OTU_15 (*Aerococcus christensenii*), OTU_16 (*P*. *amnii*), OTU_17 (*S*. *sanguinegens*), and OTU_24 (*Dialister unclassified*). Elafin correlated negatively with acetate, succinate, and pH but positively correlated with lactate and aspartate (Supplemental Figure 12A). In high-risk women (Figure 3B), cathelicidin correlated positively with HNE, corresponding with the biological corelease of cathelicidin and HNE from neutrophils. Cathelicidin was positively correlated with acetate, betaine, choline, glucose, and phenylalanine and negatively with OTU_1 (*L*. *crispatus*) (Figure 3B). Neither cathelicidin nor HNE were clearly associated with other individual OTUs in early pregnancy, but the metabolites with which they correlate are associated with PCoA group D. In high-risk women at late gestation, OTU_2 (*L*. *iners*) showed a negative correlation with cathelicidin while OTU_1 (*L*. *crispatus*) presented a positive correlation (Supplemental Figure 12B).

### Developing sPTB models using species-level phylotypes, metabolites, and HDPs.

Exploratory statistical modeling, to gain insight into which metabolites and bacteria influence risk of sPTB37 and sPTB34 prediction, was undertaken using all available data. Individual sequence reads were assigned to species-level phylotypes for this analysis.

Considering phylotypes individually for sPTB37, only *L*. *crispatus* (low relative abundance) and *L*. *gasseri* emerged as significant. However, when combined into a model, only *L*. *crispatus* remained significant and the ROC curve area for this microbiota model was poor (0.647; 95% CI, 0.590–0.704) and reduced further when stratified for Black ethnicity (0.455; 95% CI, 0.342–0.568).

Considering metabolites, stepwise regression analysis identified a composite of 7 metabolites that could predict sPTB37 (ROC curve area, 0.752; Supplemental Table 6); this included leucine, tyrosine, aspartate, lactate, betaine, acetate, and Ca^2+^. This model had reasonable ROC curve areas in relation to sampling times: 10–15^+6^ weeks (0.748); 16–23^+6^ (0.751), and also ethnicity (Black women, 0.716; White women, 0.750; Other, 0.751). The model only exhibited modest performance for sPTB34, particularly when stratifying for ethnicity (ROC curve area, 0.728 all women; white women, 0.762; black women, 0.632; other, 0.722). Addition of phylotypes or elafin to the metabolite model did not improve its performance. For sPTB34, separate unadjusted analyses identified 7 individual metabolites as statistically significant (acetate, methionine, aspartate, betaine, glucose, free EDTA, Ca^2+^). Following stepwise regression, however, only glucose, aspartate, and Ca^2+^ were retained in the model (Supplemental Table 7).

**Figure 4:**
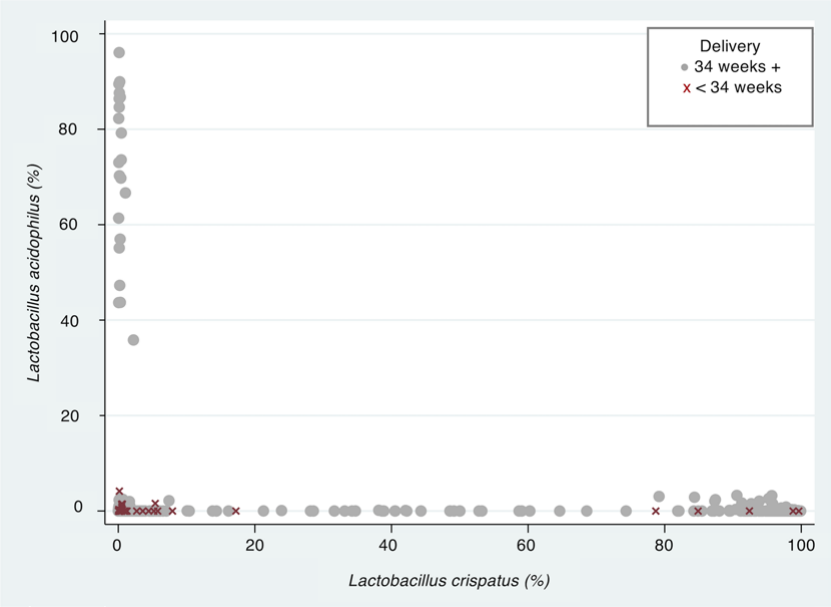
CVF *L. acidophilus* proportion above 20% is associated with term birth. Relationship between the percentage of *L*. *crispatus* and *L*. *acidophilus* in cervicovaginal fluid (CVF) of women stratified by preterm delivery < 34 weeks (sPTB34, red) or delivery > 34 weeks (gray). Data from *n =* 618 samples (10–15^+6^ and 16-23^+6^ weeks) from *n =* 341 women.

Using phylotypes alone for prediction estimates and stepwise logistic regression, low *L*. *crispatus* (odds ratio [OR], 0.196; 95% CI, 0.054– 0.714, *P <* 0.01) and *L*. *acidophilus* (OR, 0.010; 95% CI, 0.001–0.099, *P <* 0.001) were retained in the prediction model for sPTB34 weeks. However, when ethnicity were included in the model, *L*. *crispatus* became less important (Supplemental Table 8). An interesting relationship between phylotypes identified as *L*. *crispatus* and *L*. *acidophilus* also emerged (Figure 4). For women where their CVF had low relative abundance of *L*. *crispatus* but a relatively high proportion of *L*. *acidophilus* (20%), there was no sPTB34 reported.

**Figure 5:**
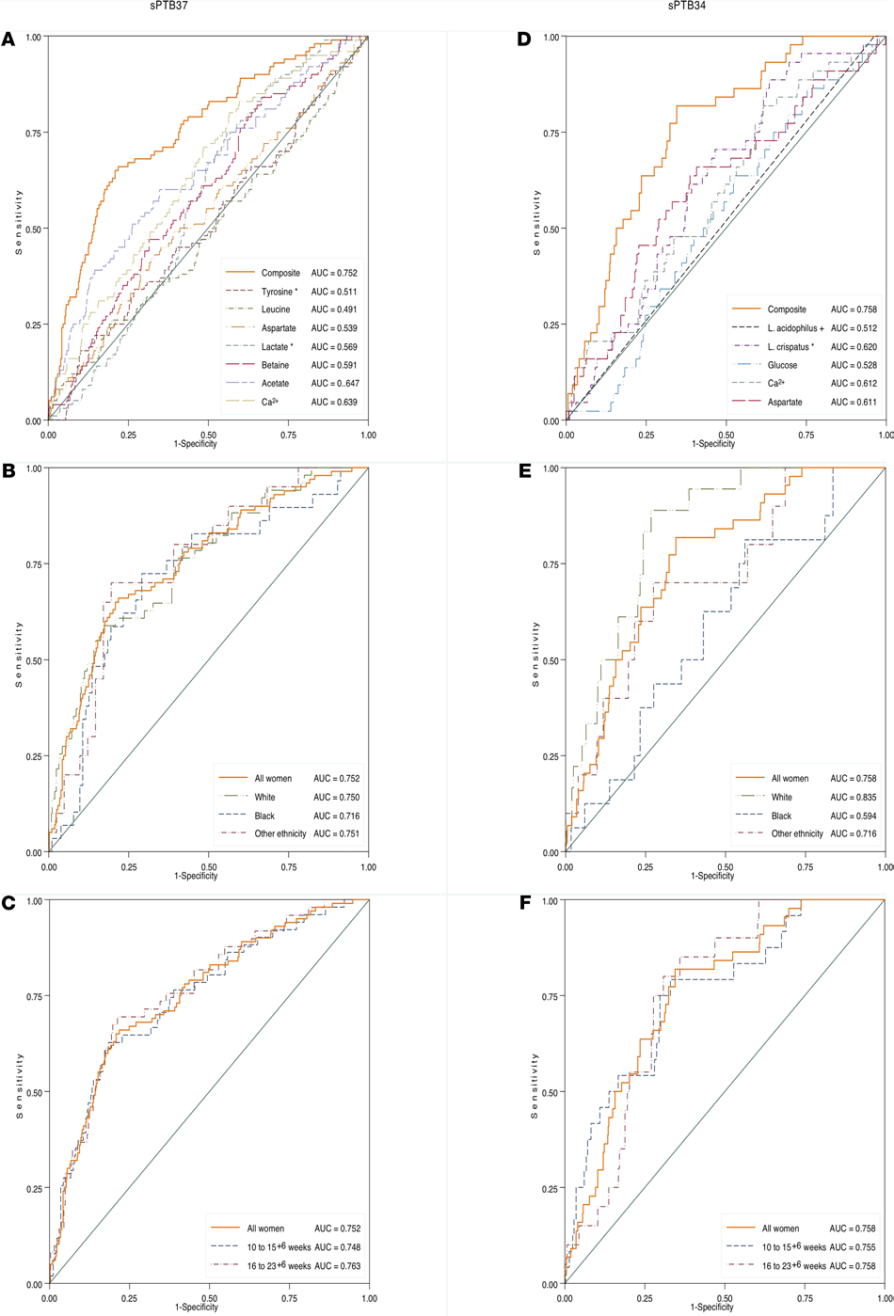
Receiver operating characteristic (ROC) curves areas and AUC for composite models for spontaneous preterm birth predictions. (**A**–**F**) sPTB37 (**A**–**C**) and sPTB34 prediction (**D**–**F**) (total number of samples, *n =* 618; *n =* 425 samples from White women, *n =* 132 from Black women, and *n =* 61 from women reporting other ethnicities; *n* = 306 from 10–15^+6^ weeks or *n =* 312 from 16–23^+4^ weeks). (**A**–**C**) a model using a composite of 7 cervicovaginal fluid (CVF) metabolites performs equally for prediction of sPTB37 when stratified by ethnicity and gestation of CVF sampling (10–15^+6^ weeks or 16–23^+4^ weeks). (**D** and **E**) For sPTB34, a model of 3 CVF metabolites and CVF *L*. *crispatus* and *L*. *acidophilus* proportions shows differences in performance when stratified by ethnicity. (**F**) sPTB34 model performs similarly when testing samples taken between 10–15^+6^ or 16–23^+6^ weeks.

We further assessed the 3-metabolite composite model by combining it with *L*. *crispatus* and *L*. *acidophilus* proportions (± elafin) to assess risk prediction for sPTB34. Both *L*. *crispatus* and *L*. *acidophilus* improved the model (Supplemental Table 9), but elafin did not. This gave a final model (adjusted for 37 potential predictors) that included *L*. *crispatus*, Ca^2+^, aspartate, *L*. *acidophilus*, and glucose. Ethnicity further modified the model (Supplemental Table 9). ROC curves are shown in Figure 5. We did not assess cathelicidin, as it was measured only in high-risk women and has been reported previously (30).

## Discussion

sPTB is a complex syndrome, and although disturbances of the cervicovaginal microbiota are increasingly implicated, any clinical benefit from these observations has yet to be realized. Consideration of how vaginal microbiota profiles may contribute to pathophysiological pathways that lead to sPTB has also been limited. To address these knowledge gaps, we explored whether increased risk of sPTB was directly associated with the cervicovaginal metabolic profile, alterations to the host response, and the presence of specific bacteria with a holistic strategy. We demonstrated that the cervicovaginal environment differs based on a woman’s obstetric history and ethnicity. We have found that a more diverse bacterial CVF profile in pregnancy is associated with raised pH, host response markers, and atypical metabolite profiles. We have also identified several OTUs and phylotypes related to term pregnancy and sPTB, with *L*. *acidophilus* (as a phylotype and specific OTU) emerging as being protective against early sPTB — a finding that presents a possible tool in the prevention of sPTB. These observations strengthen our working hypothesis that an inflammatory environment evoked by the vaginal bacteria increases the risk of inadequate cervicovaginal defense and reduced cervical integrity. Furthermore, interrogation of individual and integrated data sets has given insight into functional correlations between bacterial groups and both metabolic and immune response activity (microbial/host interactions), and it has enabled exploratory statistical modeling of CVF risk factors for sPTB. Our approach has highlighted the potential for measures of biological variables in CVF to improve sPTB risk stratification and subsequent intervention.

Taking into account that racial and ethnic differences could also reflect differences in environmental and social exposures, our study is in agreement with reports that Black ethnicity is a risk factor for sPTB (31–33), and it complements and adds knowledge to findings from previous studies (9, 10, 12, 16, 17, 19, 25, 26, 34, 35) by demonstrating differences between the cervicovaginal environments based on women self-reporting White and Black racial backgrounds and providing data on the whole cervicovaginal environment (9, 10, 12, 16, 17, 19, 25, 26). A healthy vaginal environment in pregnancy is considered to be one with low microbial diversity (14, 25). Correspondingly, we also identified OTU_1 (*L*. *crispatus*) to be associated with low risk of sPTB (15–17, 21, 36). Notably, when stratified for ethnicity, this relationship was stronger for White than Black women. OTU_6 (*L*. *acidophilus)* provided protection against sPTB34 in all women, even when aggregating data as phylotypes. Therefore, early pregnancy screening for both species has promise as a much-needed screening tool for risk stratification for women in their first pregnancy when their risk is unknown. For women already identified as high-risk, based on obstetric history, such testing could provide reassurance of vaginal health, insight into their specific pathophysiology, and indicators for treatment. We are currently validating the clinical utility of this approach in our cohort.

The PCoA groups, which were identified in our heterogenous UK cohort, were consistent with, but had some differences to, those previously published (13, 17, 37). For example, our data do not fully support the previous inference that a *L*. *iners*–dominated CST confers the greatest risk of sPTB (11, 36). This may be due to acknowledged differences in methodology (14, 38) or limited scope to stratify by ethnicity. In contrast, *L*. *jensenii*, which in other studies clearly defines CST V (14, 17), was found in high abundance in some of the samples belonging to PCoA groups C and D, but the abundance of *L*. *jensenii* was not a major discriminant between groups. Similarly, *L*. *gasseri* was the major species found in PCoA group B. In our study, 10% of samples fell into group B, while Ravel et al. and MacIntyre et al., respectively, reported that 6.3% and 9% of samples fell into the corresponding CST II (14, 17). The relative abundance of *L*. *gasseri* was, therefore, not different to that previously described.

Differences with previous studies could be also be influenced by the use of modified PCR primers to amplify the 16S rRNA gene and improve detection of *Bifidobacteriaceae*, including *G. vaginalis group,* often missed with unmodified V1–V2 primers (14, 39). Limited detection of *G*. *vaginalis group* in other data sets could shift CST classifications and overestimate the importance of *L*. *iners* (40). The majority of Black women in our cohort exhibited high levels of *L*. *iners* (OTU_2), but only 24% of pregnancies resulted in sPTB, similar to a study of predominantly Black American women (10). Indeed, our statistical model (whole cohort and ethnicity stratification) rejected *L*. *iners* as a good predictor of sPTB risk.

Classification of women by PCoA groups in early pregnancy, even when stratified by ethnicity, also did not accurately identify those who delivered prematurely. Discovery of a number of OTUs that correlate with sPTB suggest that developing a “perfect” CVF prediction test based on a single bacterial species will be challenging, but the identification of a panel of candidate bacteria from women at risk could be of some value; this is a concept currently being pursued by many researchers and commercial organizations (41).

The need for a panel of specific bacteria to identify sPTB risk could be bypassed by evaluating the metabolic profile defined by the vaginal bacterial community (42–44). The microbiota products D- and L-lactate, acetate, and succinate have been proposed as useful predictors of sPTB (14, 15, 21, 42). However, few studies have considered the spectrum of CVF metabolites in relation to sPTB.

By integrating data sets, we identified distinct associations between PCoA groups and metabolites. For example, the positive correlation of glucose with PCoA groups C and D reflected the low relative abundance of *L*. *crispatus*, a major consumer of this sugar. Betaine, choline, and carnitine, involved in the trimethylamine synthesis pathway (45), were also higher in PCoA groups C and D compared with *L*. *crispatus–*dominated PCoA.

Raised acetate and Ca^2+^ levels in early-pregnancy CVF were associated with sPTB delivery. Acetate, a marker of anaerobic fermentation, has previously been related to sPTB and inflammation (15, 44, 46, 47) via cervicovaginal epithelial cell cytokine release (47). The relationship with Ca^2+^ is potentially novel and unexplained, although increased Ca^2+^ extrusion from host cells or bacteria could play a role. In other tissues, raised extracellular Ca^2+^ concentration can influence Ca^2+^ sensing receptor signaling pathways associated with inflammation and epithelial barrier integrity (48).

Metabolite-OTU correlations also revealed information about the coexistence of communities, since not all OTUs assumed to be functionally associated showed similar correlations with metabolites. Many BV-associated bacterial OTUs, for example, were positively associated with Ca^2+^ and acetate, but diverged with regard to betaine, formate, and succinate.

Aspartate, a carbon source for a number of anaerobic bacteria, was included in our statistical models for identification of women at higher risk of preterm birth. The involvement of aspartate appears complex, since — while being positively correlated with OTU_6 (*L*. *acidophilus*), which our data suggest is protective against sPTB34 — it is also positively correlated with 2 other OTUs, OTU_21 (*B*. *breve*) and OTU_27 (*L*. *delbrueckii*), which were frequently associated with sPTB in our analysis. Given neither of these 2 OTUs correlated with OTU_6 (*L*. *acidophilus*), we hypothesize that any role they may play in relation to sPTB only emerges in the absence of OTU_6 (*L*. *acidophilus*); this will require further validation.

Host response markers, elafin, cathelicidin, and HNE, provided insight into the cervicovaginal inflammatory milieu. We recently published detailed profiles of these proteins in relation to sPTB (30) and in the present subanalysis report that both elafin and cathelicidin were higher in high-risk Black women compared with White women. Independent of ethnicity, cathelicidin concentrations were clearly affected by the resident bacterial community (PCoA groups and OTUs). While elafin showed some association with PCoA groups and 2 OTUs, there were striking correlations with metabolites (positively with lactate, aspartate, and leucine; negatively with Ca^2+^, acetate, and pH). This suggests that elafin is a marker of vaginal health regulated by metabolic/inflammatory moieties rather than specific bacteria. Similarly, neither HNE or cathelicidin were strongly associated with specific OTUs, but rather with metabolites (e.g., cathelicidin was associated with Ca^2+^, formate, betaine, methionine, and acetate), suggesting that their neutrophil/epithelial release or expression (29) is regulated by metabolites or indirectly by pH and/or inflammatory mediators (e.g., epithelial cytokines; ref. 47). These data reinforce the suggested importance of HDPs in maintenance of a healthy vaginal environment (49, 50).

The relationship of the vaginal environment with sPTB was further investigated by building statistical models, aiming to inform biological understanding and to signpost avenues for future biomarker development.

For sPTB37, a composite model of metabolites alone was the best predictor. Since this model performed equally well for women of different ethnicities and at both gestational sampling points, this mix of metabolites associated with vaginal health and dysbiosis appears to provide a robust functional readout of complex cervicovaginal bacterial communities.

For sPTB34, a simpler model comprising glucose, aspartate and Ca^2+^ plus inclusion of *L*. *crispatus* and *L*. *acidophilus* proportions was most useful. Although metabolites were similar in both models, the sPTB34 model performed less well when stratified by ethnicity, presumably influenced by the inclusion of *L*. *crispatus* (related to term outcome in White women). The relative abundance of *L*. *acidophilus* phylotype also showed predictive potential when considered alone; we suggest that *L*. *acidophilus* was sufficient to protect against sPTB34, perhaps filling a niche created when *L*. *crispatus* was less abundant. This finding, which emerged consistently in different analyses, suggests that women with a more diverse vaginal microbiome community benefit from the coexistence of *L*. *acidophilus*. It follows that *L*. *acidophilus* as a probiotic supplement, if appropriately targeted, could improve pregnancy outcome.

The strength of repeat testing using both in the first and second trimester samples was explored, but it did not significantly improve prediction. Indeed, in clinical practice, there is greater benefit in focusing on developing predictive tests that can be used early in pregnancy to identify women as high risk in order to inform prophylactic intervention. Third-trimester measurements, which were not performed here, might have potential to inform understanding of preterm labor, but measuring at this time point would not allow time for interventions aimed at reducing risk.

A greater proportion of women who developed a short cervix by < 24 weeks of pregnancy were also classified in the PCoA group C (*L*. *iners*), similar to the findings of Kindinger et al. (11). In contrast, Gerson et al. (51) have suggested that cervical shortening with subsequent sPTB is associated with a more mixed anaerobic bacterial community. Nevertheless, this does not rule out the possibility that restructuring of the cervical tissue may occur through pathways unrelated to the influences of the microbiota.

This study had several strengths and limitations — in particular, our multi–data type interrogation of the vaginal environment (combining vaginal microbiota, metabolome, and host defense peptides) and the use of statistical modeling to identity potential tests for prediction of preterm birth. Limitations include 16S rRNA gene sequencing for identification of bacteria species; this could be supplemented with functional analyses to identify and understand aspartate-consuming and succinate-producing anaerobes. Similarly, the contributions of other microorganisms such as viruses, archaea, protozoa, and fungi were not included. Our study was also limited by a relatively low number of Black women and Asian women (who fall into our “Other” category) compared with White women, although the percentage of Black women in our study was greater (21.7%) that then the UK population national average (3.3%; ref. 31). This did not allow us to refine our analysis based on ancestry; indeed, the use of self-reported ethnicity in the absence of genotyping may just be a surrogate for other influencing factors. These data would be strengthened by the inclusion of data relating to diet, environment, and social stressors. We did not distinguish between L- and D-lactate due to use of the NMR platform. Expanding the range of metabolites using mass spectrometry would be desirable (52), although through CVF NMR analysis, we identified 29 metabolites that are comparable with the numbers identified in previous NMR studies — i.e., 6 (42), 11 (46), and 28 (44).

To further explore the relationship between cervical shortening and the vaginal environment, larger studies are required that can fully address the issue of ethnicity and the use of different interventions (53).

In summary, integration of metabolite and bacterial community composition has significant potential for enhancing our understanding of the contribution of the vaginal environment to sPTB. We have developed statistical models that suggest that *L*. *acidophilus* may be a potential probiotic to reduce risk of sPTB. It is vital to consider the influence of ethnic origin in tandem with women’s environmental and social exposures on the relationship between vaginal environment and sPTB and to consider replicating studies in low- and middle-income countries where the burden of sPTB is highest. Finally, this study shows the importance of precision medicine and the need to implement novel tools for data integration to better understand the complexity of diseases such as sPTB.

## Methods

### Participant and sample collection.

Participants (*n =* 353) for this study are a subgroup selected from an ongoing pregnancy cohort study (INSIGHT; ref. 30), a prospective longitudinal observational study of women at high and low risk of sPTB. High-risk women (1 or more of prior sPTB or late miscarriage between 16 and 37 weeks’ gestation, previous destructive cervical surgery, uterine anomaly, or incidental finding of a cervical length < 25 mm on transvaginal ultrasound scan) were recruited from high-risk antenatal clinics in 4 UK tertiary hospitals. Low-risk women were recruited from the general antenatal population at their dating ultrasound appointment (10–13^+6^ weeks).

During speculum examination, CVF was obtained from the posterior fornix, using a Dacron swab (for metabolite and host defense peptide analysis) and then inserted into 750 μL of standard phosphate-buffered saline solution containing protease inhibitors and EDTA (Complete, Roche Diagnostics GmbH; ref. 30). Cell-free supernatants were divided into aliquots (~110 μL) and stored at –80°C until analysis. A second nylon flocked swab (Copan eSwab, VWR International Ltd.) was obtained for 16S analysis, placed into 1 mL of TE buffer (Promega), and transported immediately on ice to the laboratory and stored at –80°C until analysis.

Pregnancy outcome data were collected and monitored from case note review. sPTB was identified if women had a spontaneous onset of labor or have experienced premature rupture of membranes and delivered prior to 37 weeks of gestation (this included spontaneous late miscarriages > 16 weeks). IUD pregnancies and iatrogenic deliveries were not included in the analyses.

### Cervical length measurement.

Cervical length measurement by transvaginal ultrasound scan was performed by trained operators in accordance with standardized protocols (at least once between 14 and 24 weeks, usually at every clinical visit). The total closed length was measured 3 times (mm) with the shortest measurement recorded. For analysis purpose, the cervix was classified as “short” if it measured below 25 mm prior to 24^+0^ weeks of gestation.

### NMR.

CVF samples (described above) were immersed in liquid nitrogen, lyophilized at −58°C overnight, and resuspended in 550 μL D_2_O. CVF metabolite profiles were generated using ^1^H-NMR spectra acquisition, processing, and OPLS-DA, done as reported previously (54). For this study, spectral regions above 8.5 ppm and below 0.5 ppm were excluded for noise content. The water peak and trimethylsilylpropanoic acid reference signals were also excluded. A total of 29 metabolites were identified using the Chenomx NMR suite software (Chenomx Inc.). The signal from propylene glycol was removed from analyses, as it is a known contaminant from the gel used in transvaginal scanning.

### Bacterial community analysis.

DNA was extracted from thawed samples using the GenElute Bacterial Genomic DNA kit (Sigma-Aldrich), modified to optimize lysis of Gram-positive bacteria. From each DNA extract, variable regions V1–V2 of the 16S rRNA gene were amplified by PCR using fusion primers incorporating template specific primers, MiSeq adapters, and barcodes to achieve a double indexing system. The forward primer 27F included the YM modification (55) to improve recovery of the family *Bifidobacteriaceae*, including *G. vaginalis group*. The specific primer sequences were: 27F-YM (AGAGTTTGATYMTGGCTCAG) and 338R-R (TGCTGCCTCCCGTAGRAGT). Amplicons were purified and normalized using the SequalPrep Normalization Plate Kit (Thermo Fisher Scientific). Sequencing was performed at the Barts and The London Genome Centre using Illumina MiSeq 2 × 250 flow cell paired-end sequencing. Sequence reactions were spiked with 10% 12.5 pM PhiX DNA. Reads were filtered by quality score using the fastqPairedFilter command of DADA2 R package (56) to remove sequences with an expected error over 2 bp. Forward and reverse sequences were truncated at 250 and 200 bp, respectively.

Filtered sequences were analyzed using mothur (version 1.36.1) SOP (57). Sequences were clustered into OTUs at a sequence dissimilarity distance of 0.015 using the opticlust algorithm. Consensus identification of OTUs was performed with reference to the Vaginal 16S rDNA Reference Database (58).

Inspection of the sequences from the negative control samples revealed *Pseudomonas gessardii* as a reagent contaminant, and all related OTUs were removed prior to analysis.

For the comparisons between microbiome and metabolome, a normalized version of the OTU table was used where reads for each sample were rescaled to a depth of 3570. A thetaYC dissimilarity matrix was generated using mothur, by iterating 1000 times the subsampling at a depth of 3570 sequences. This matrix was used to generate PCoA coordinates that were plotted using the ggplot2 package. The PCoA groups A, B, C, D, and E were identified on the plot, and the samples were classified by group based on the values of their 2 principal coordinates. To compare the relative proportions of bacterial species of interest, a phylotype analysis was performed, identifying individual sequences to species level by means of the mothur classify.seqs command. Sample composition at species level is provided in Supplemental Table 10.

### Measurement of antimicrobial peptides/proteins.

As previously published (30), samples were thawed at room temperature, briefly vortexed, and analyzed by ELISA (Trappin2/elafin, HK318; cathelicidin [LL37], HK321; HNE, HK319-02; Hycult Biotech) in duplicate, according to manufacturer’s instructions. Samples for elafin measurement were diluted in sample buffer (1:20 and 1:100 for each sample) to ensure positioning within the standard curve, based on results obtained from a pilot study (27). Samples for HNE measurement were diluted in sample buffer 1:200 for each sample. CVF samples for cathelicidin measurement were undiluted. Intraassay variability was < 15%, based on a pooled CVF sample (random set of 10 CVF samples included on each plate). Final concentrations were calculated from the standard curves using logistic regression. Accepted coefficient of variability (CV) between sample duplicates was < 20%. The elafin concentration used in the statistical analysis of host-defense peptides was a derived value based on the 2 dilutions, to allow for dilutional effect (59).

### Data and materials availability.

The 16S rRNA gene sequence data from this study have been deposited with the NCBI SRA as accession PRJNA660627.

### Statistics.

Statistical analyses on processed metabolites and normalized OTUs were performed in R v3.6.1. The α diversity was estimated based on Shannon (60) and Inverse Simpson (61) index with pairwise comparison by Wilcoxon signed-rank based on these indexes, indicated as *P*_Shannon_ and *P*_InverseSimpson_ (packages *Phyloseq* and *Vegan*). PERMANOVA was estimated using the Bray Curtis distance matrix. Spearman’s correlation analyses were performed using the function rcorr.adjust from the RcmdrMisc package with method “spearman” and “complete.obs”; *P* values were calculated using FDR correction (62). LEfSe (63) on the normalized, rescaled OTU table was performed; *P* < 0.05 and a score > 3.0 were considered significant. Wilcoxon rank-sum and Kruskal-Wallis test were performed for sample comparisons, *P* values identified via these tests are annotated as *P*_Wilcoxon_ and *P*_Kruskal-Wallis_, respectively. The *ropls* package was used for OPLS-DA analyses of metabolites whilst *Rvolcano* was selected to generate fold changes and volcano plots. Feature selection analysis was performed using the univariate Cox proportional hazards model (ref. 64; within the *CancerSubtypes* package) with metabolome data normalized by *Z* score, gestation delivery in days, and delivery outcome as event (0 for term and 1 for sPTB37 or sPTB34).

Prediction analyses were conducted in Stata versions 15 and 16 (StataCorp.). Cervicovaginal phylotypes (*L*. *crispatus*, *L*. *acidophilus*, *S*. *amnii*, *A*. *vaginae*, *G*. *vaginalis group*, *L*. *gasseri*, *L*. *jensenii*, *L*. *iners*, Megasphaera “OTU 70”) plus all other classifications, which made up *<* 0.1, obtained in either early or late samples were used for prediction modelling.

Logistic regression was used to determine subsets of the microbiome and metabolome significantly associated with the outcome. For NMR metabolites, both logged and unlogged values of the metabolome were tested. However, the only significant relationships were found using unlogged values. For each of microbiota, metabolites, and HDP, stepwise logistic regression with probability of entry set at *P* < 0.05 was used to develop a prediction model for sPTB34 and sPTB37. The performance of the resulting models was compared with its components using ROC curves areas. Differences in performance by gestation of test and ethnicity (3 groups) were likewise investigated (65).

### Study approval.

The INSIGHT study was approved through by the NHS Human Research Authority (HRA), London – City and East Research Ethics Committee (13/LO/1393). Informed written consent was obtained from all participants.

## Author contributions

RMT conceived the presented the idea. RMT, NLH, and AJM contributed to study design. NLH, AHS, and AER recruited for the study and collected samples and clinical data. NLH, ECS, AER, EMP, WGW, TK, and VM undertook laboratory analyses. FF, TK, EMP, PTS, RMT, NLH, DKVM, KD, and WGW undertook statistical analysis. RMT, FF, and AJM interpreted the data. FF and RMT wrote the manuscript, with help from AJM. All authors reviewed and provided further input into the final version of the manuscript.

## Supplementary Material

Supplemental data

Supplemental table 10

## Figures and Tables

**Table 1:**
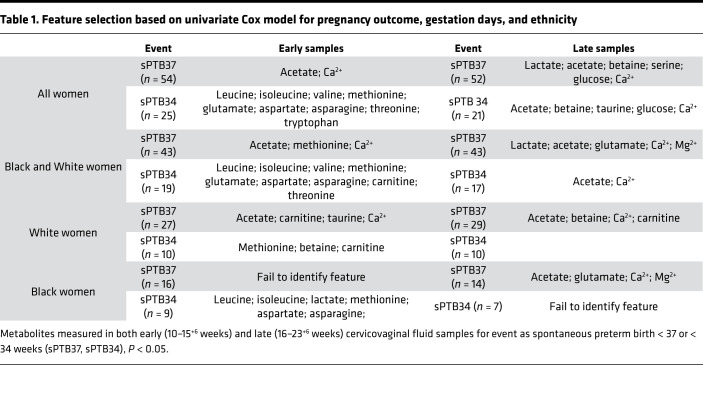
Feature selection based on univariate Cox model for pregnancy outcome, gestation days, and ethnicity

## Version 1 07/13/2021

In-Press Preview

## Version 2 08/23/2021

Electronic publication
