# Supplementary material for: Cervicovaginal microbiota and metabolome predict preterm birth risk in an ethnically diverse cohort
Source: JCI Insight. 2021 Aug 23;6(16):e149257. doi: 10.1172/jci.insight.149257 (PMC8410012; doi:10.1172/jci.insight.149257)
Supplement: Supplemental data [file jciinsight-6-149257-s085.pdf]

Supplementary Materials

File includes:

Figure S1: Flow and demographic of the subset of INSIGHT cohort participant used in this study

Figure S2: Exploration of BMI in relation to relation to term and preterm (<37 weeks) birth outcome and Ethnicity

Figure S3: Principal Component Analyses (PCoA) for the microbial composition of cervicovaginal fluid.

Figure S4: Heatmap representing species-level phylotypes relative abundance in each cervicovaginal fluid sample identified by 16s rRNA gene amplicon sequencing.

Figure S5: Volcano plot showing fold changes in cervicovaginal fluid metabolites.

Figure S6: Cervicovaginal fluid microbial composition based on principal component analyses (PCoA) groups in late pregnancy samples based on delivery outcome.

Figure S7: Cervicovaginal fluid microbial composition based on principal component analyses (PCoA) groups based on high-risk participant in relation to cervical length.

Figure S8: Cervicovaginal microbial distribution based on principal component analyses (PCoA) groups in relation to self-reported ethnicity and pregnancy outcome.

Figure S9: Alpha diversity analyses at OTU level of species richness within cervicovaginal fluid

Figure S10: Venn Diagram of cervicovaginal fluid OTUs composition

Figure S11: Spearman Correlation analyses of cervicovaginal fluid OTUs, metabolites and host response proteins in late samples (16-23^{+6} weeks gestation).

Figure S12: Spearman Correlation analyses of cervicovaginal fluid from whole cohort to include pH

Table S1: Participant demographics

Table S2: Exploration of cervicovaginal fluid (CVF) components

Table S3: Orthogonal Projections to Latent Structures – Discriminant Analysis (OPLS-DA) of cervicovaginal fluid metabolites differences

Table S4: LDA (Linear Discriminant Analysis) Effect Size (LEfSe) of cervicovaginal fluid OTUs in relation to term and preterm (<37 weeks) birth

Table S5: LDA (Linear Discriminant Analysis) Effect Size (LEfSe) of cervicovaginal fluid OTUs in relation to term and preterm (<34 weeks) birth

Table S6: Composite metabolite prediction model for spontaneous preterm birth <37 weeks

Table S7: Logistic regression of significant cervicovaginal three metabolites (standardized) to predict spontaneous preterm birth <34 weeks (sPTB34)

Table S8: Stepwise logistic regression model of phylotypes

Table S9: Logistic regression metabolites and phylotypes for spontaneous preterm birth <34 weeks (sPTB34)

Table S10: Characterization of the 16S microbiome based on species-level composition by samples

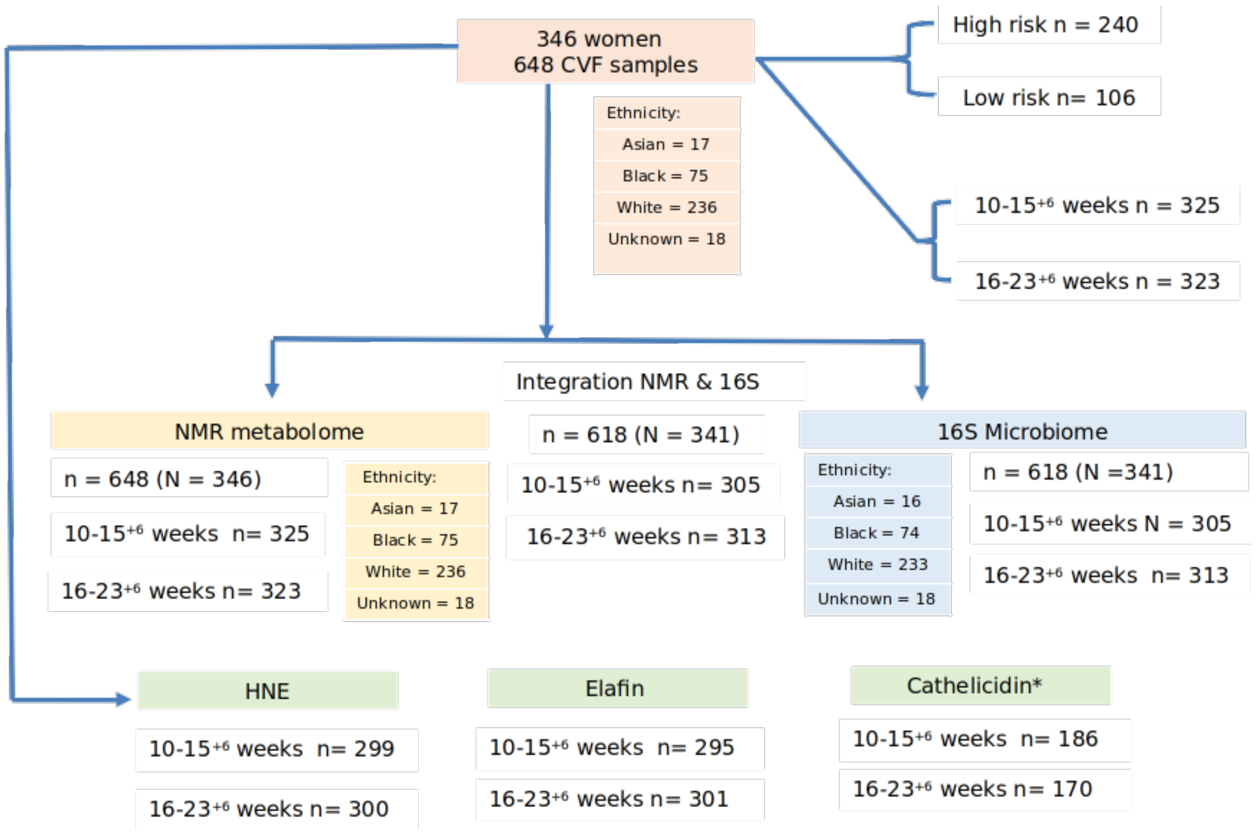

Figure S1: Flow and demographic of the subset of INSIGHT cohort participant used in this study. Data show information for women providing at least one cervicovaginal fluid sample independent of the time point. Asian and unknown ethnic groups are combined for reporting some of the analyses as 'other'. NMR: Nuclear Magnetic Resonance. CVF: cervicovaginal fluid. 16S microbiome: 16S rRNA amplicon sequencing. N: number of patients; n: number of samples. HNE: Human Neutrophil elastase. (*) indicate that Cathelicidin samples were collected only for high- risk women.

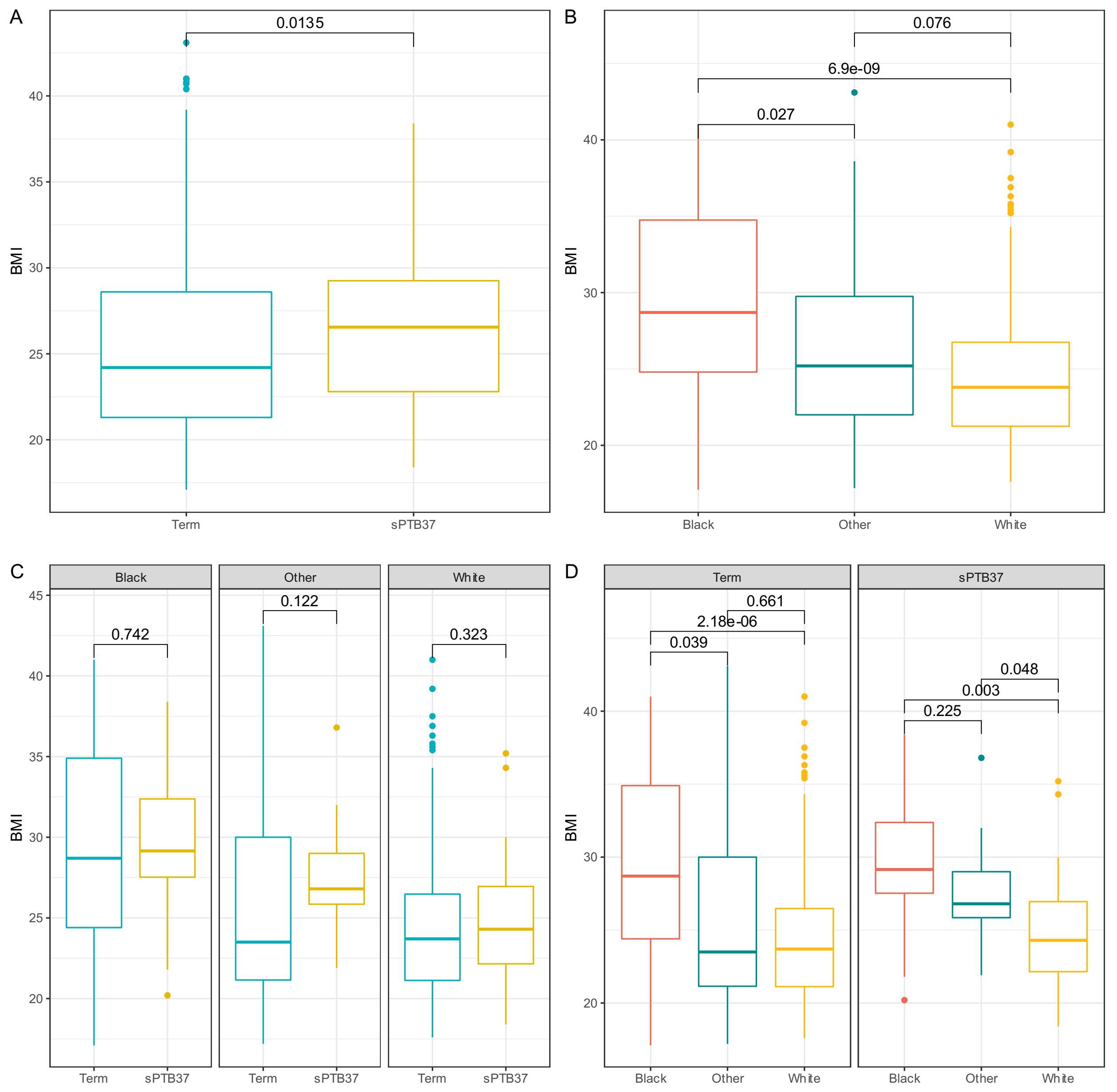

Figure S2: Exploration of BMI in relation to relation to term and preterm (<37 weeks) birth outcome and Ethnicity. BMI measured at study entry (N= 345) in relation to (a) pregnancy outcome before 37 weeks' gestation (sPTB37) and (b) to self-reported ethnicity. (c) BMI comparison with pregnancy outcome segregated by self-reported ethnicity and (d) BMI comparison with self-reported ethnicity segregated by pregnancy outcome. Wilcoxon test with p-value adjusted using false discovery rate (FDR).

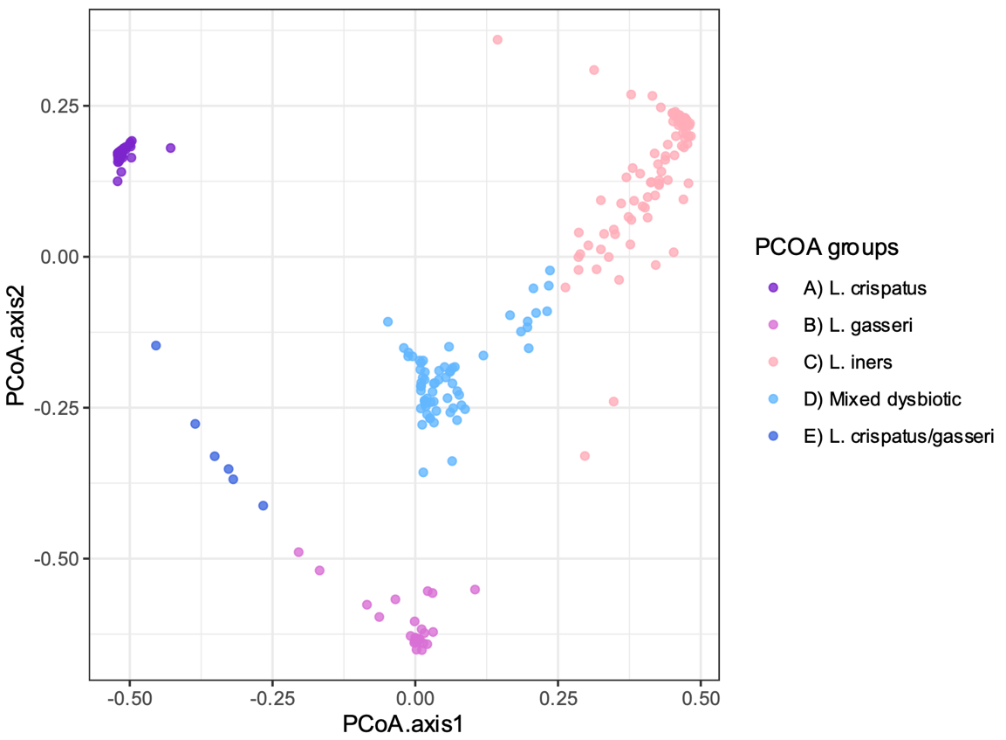

Figure S3: Principal Component Analyses (PCoA) for the microbial composition of cervicovaginal fluid. Groups distribution in the whole community in early pregnancy samples collected between 10-15^{+6} weeks gestation.

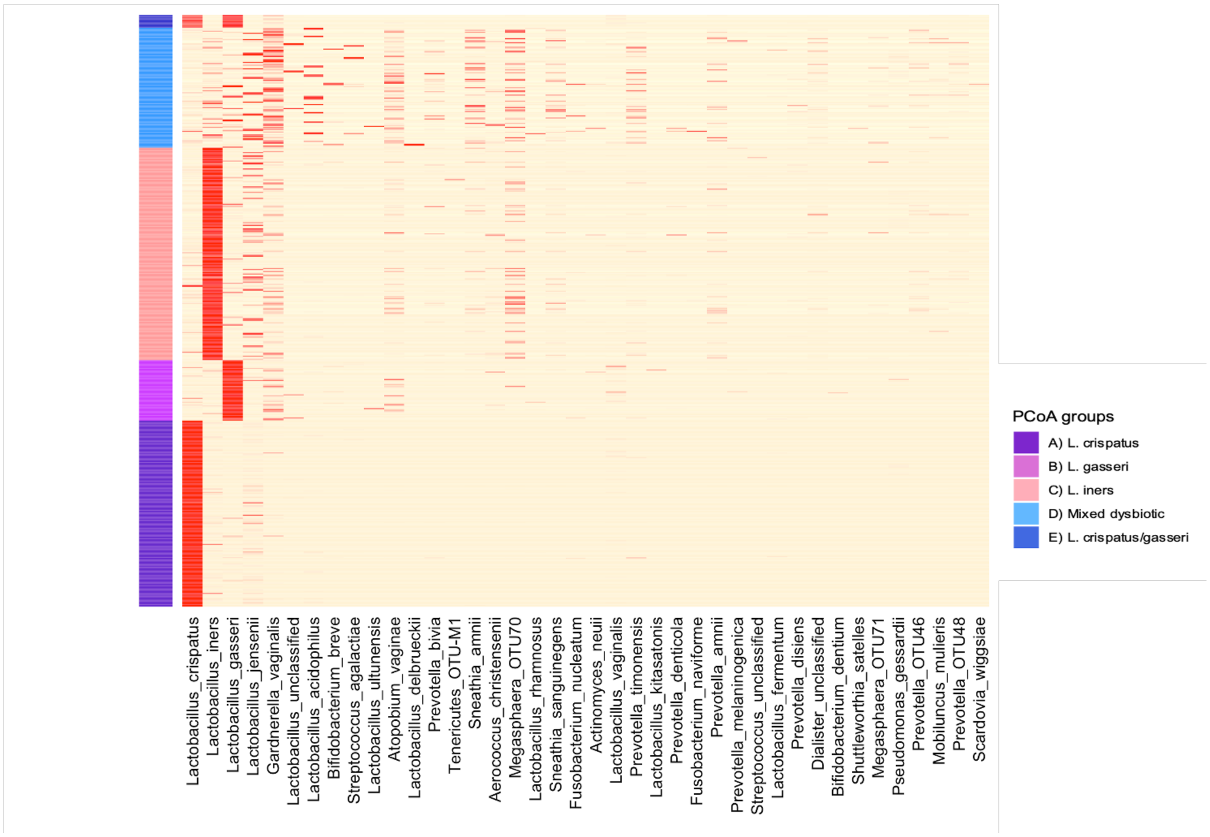

Figure S4: Heatmap representing species-level phylotypes relative abundance in each cervicovaginal fluid sample identified by 16s rRNA gene amplicon sequencing. Samples are represented on the vertical axis and phylotypes on the horizontal axis. Color scale goes from red (highest abundance) to pale yellow (absence). The colored bar on the left side identifies the samples belonging to each principal component analyses (PCoA) group.

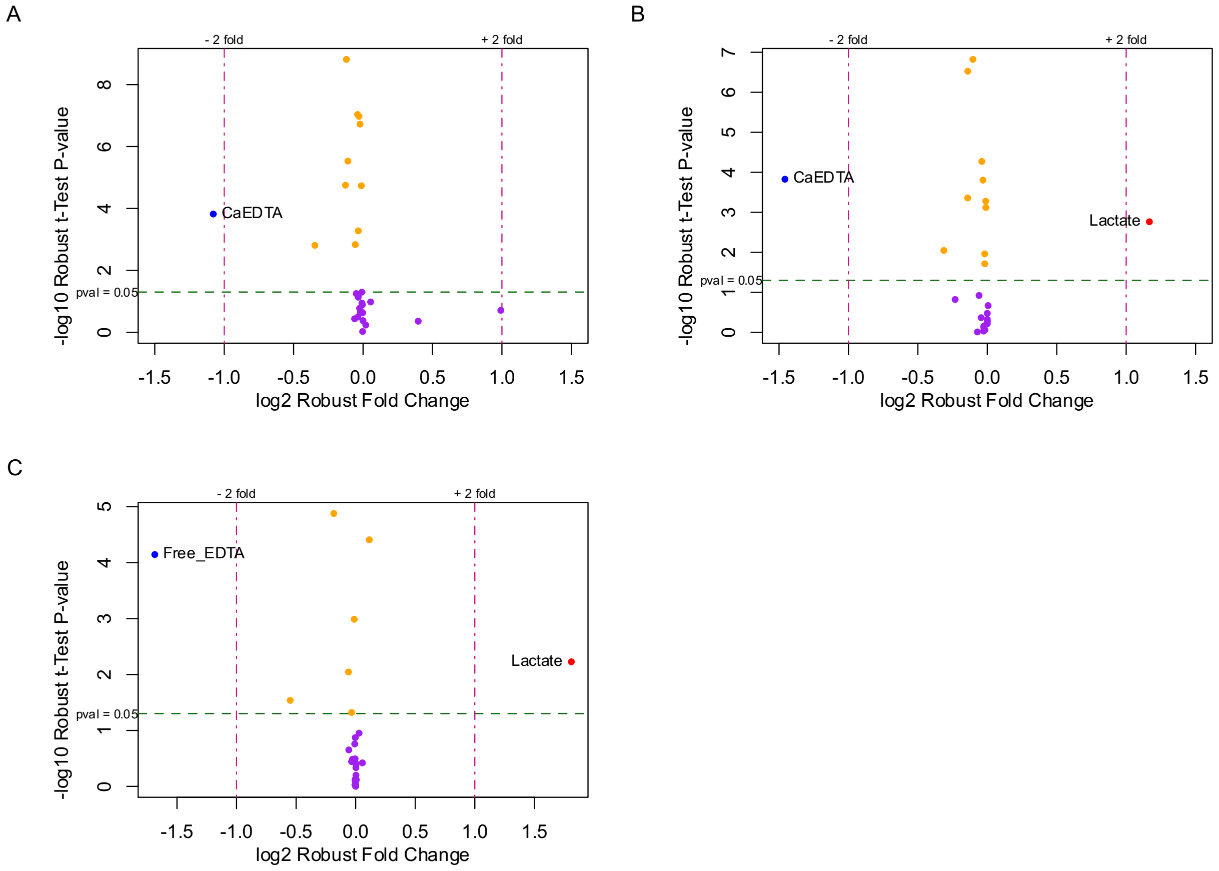

Figure S5: Volcano plot showing fold changes in cervicovaginal fluid metabolites. (A-B) Ethnicity comparison for White and Black women in early (A) and late (B) cervicovaginal fluid samples (baseline white women). Early samples comprised of N=72 Black and N=219 White women. Late samples characterized by N=70 Black and N=222 White women. (C) Comparison between low risk (N= 94) and high-risk (N=198) women in the late samples for Black and White women (baseline low-risk women).

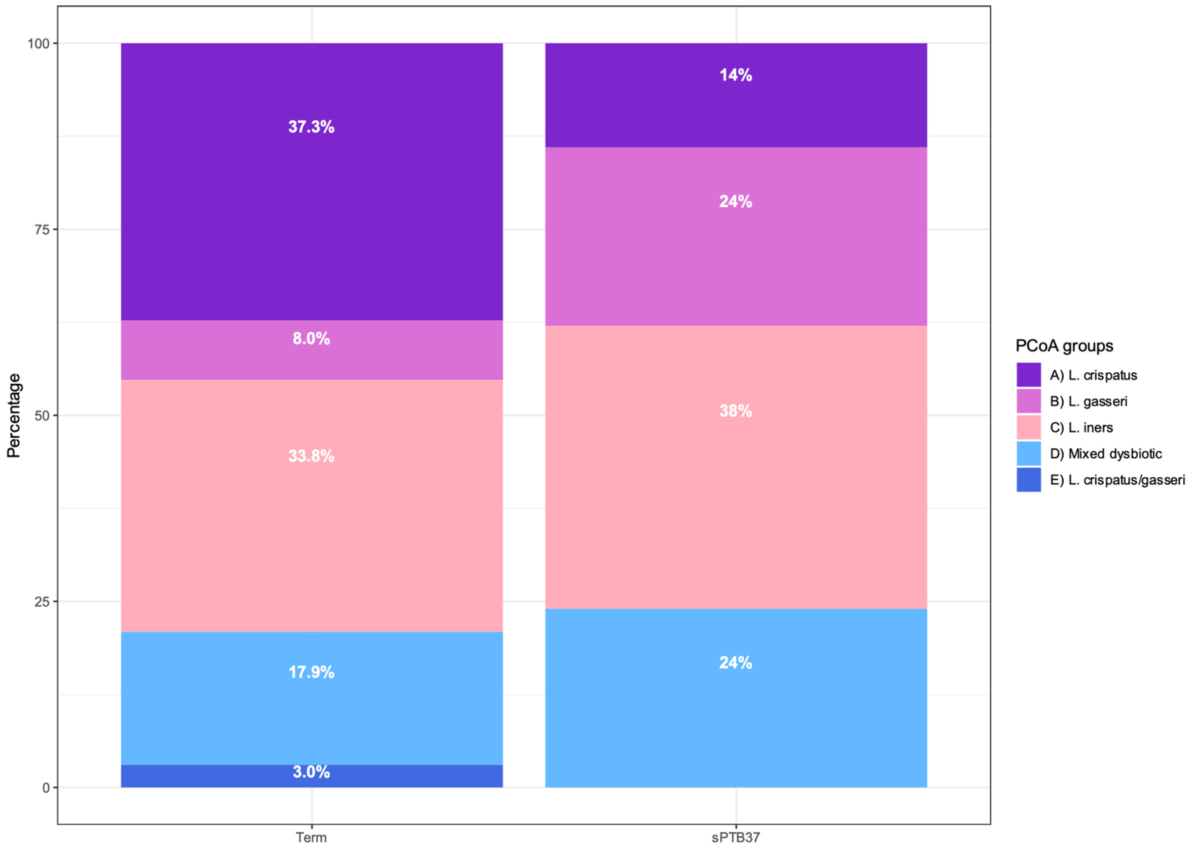

Figure S6: Cervicovaginal fluid microbial composition based on principal component analyses (PCoA) groups in late pregnancy samples based on delivery outcome. Distribution of PCoA groups in the late samples (16-23^{+6} weeks gestation) in the whole community based on delivery outcome for term and spontaneous preterm (sPTB <37 weeks) birth.

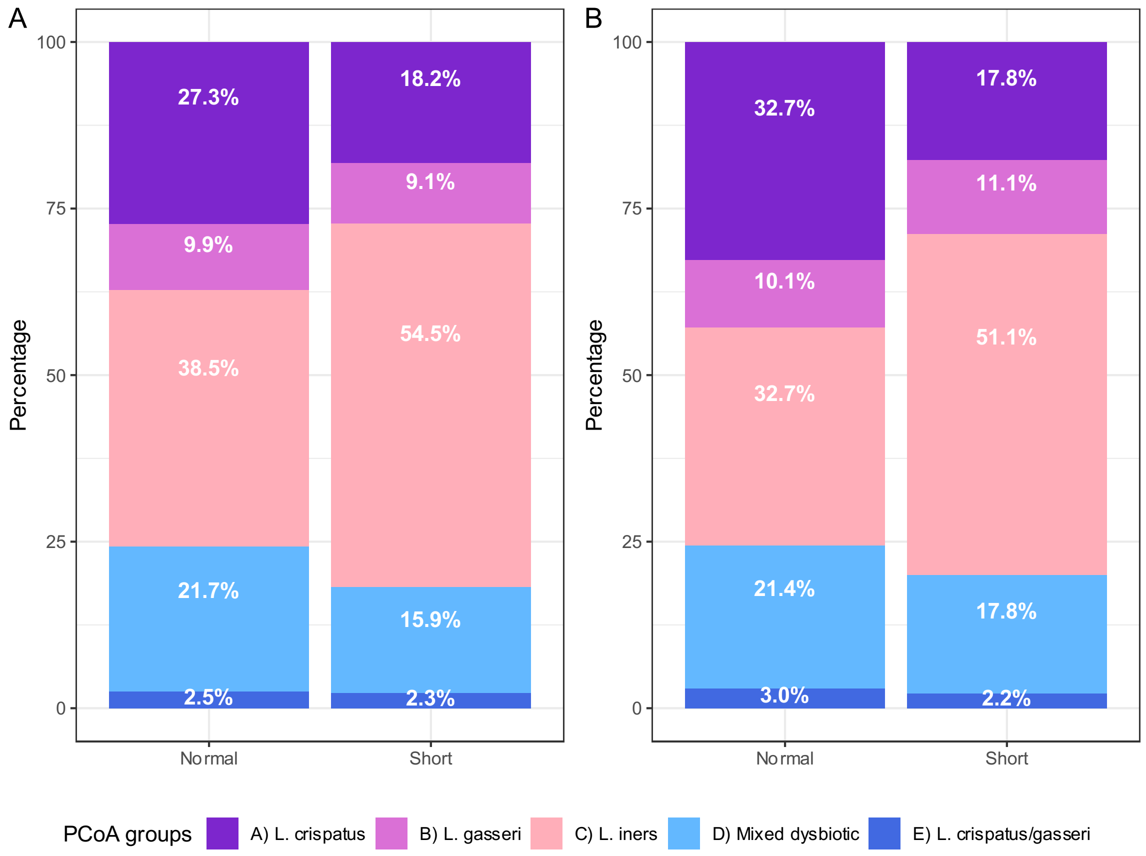

Figure S7: Cervicovaginal fluid microbial composition based on principal component analyses (PCoA) groups based on high-risk participant in relation to cervical length. High-risk women stratified into two groups, those who have a normal cervical length measured by transvaginal ultrasound versus women who develop a short cervix (<25 mm) by 24 weeks of gestation. A) early samples (10-15^{+6} weeks gestation) and B) late samples (16-23^{+6} weeks gestation). Early samples high-risk N=220 of which 45 develop a short cervix; late samples high-risk N= 219 of which 45 develop a short cervix.

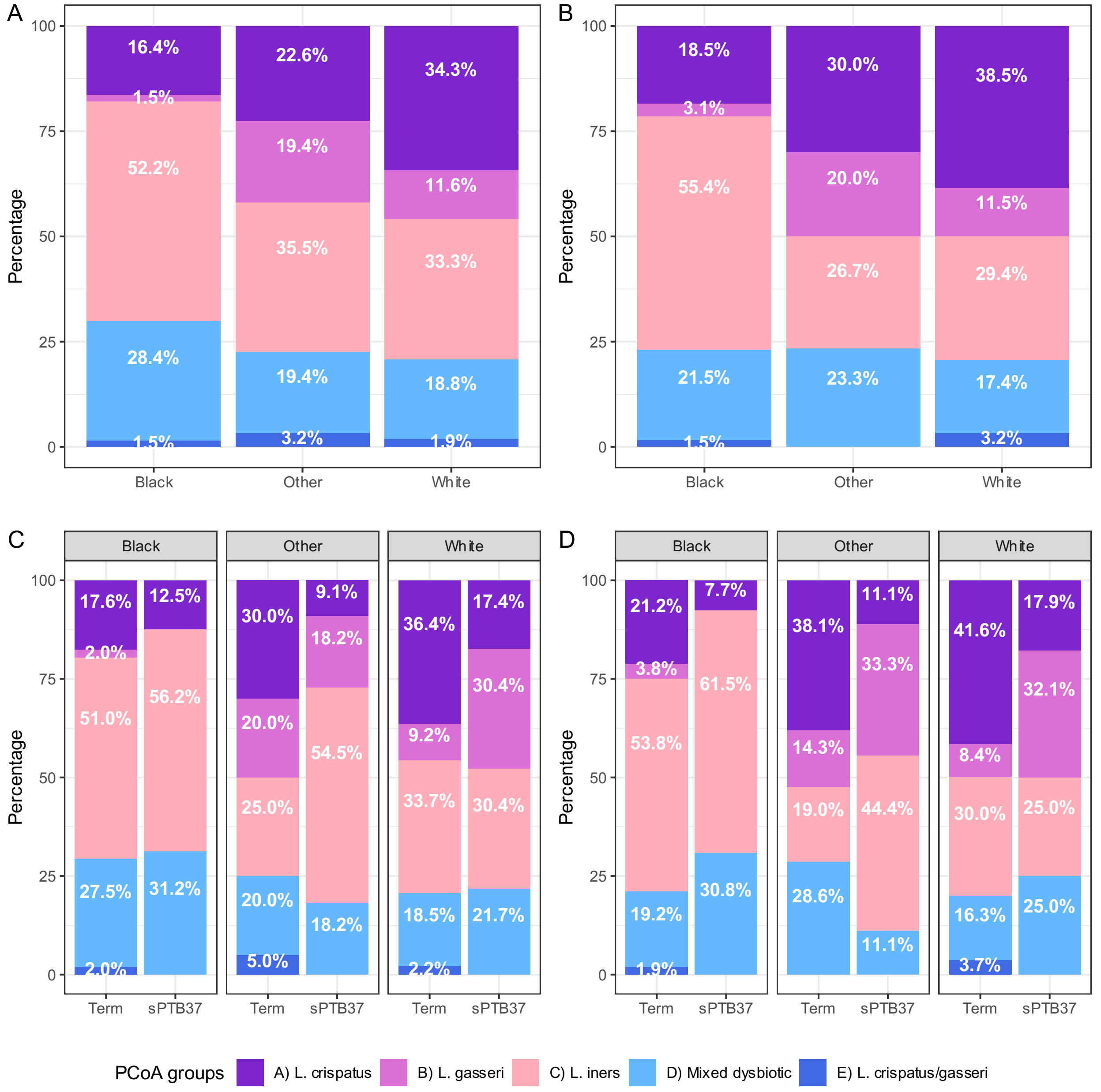

Figure S8: Cervicovaginal microbial distribution based on principal component analyses (PCoA) groups in relation to self-reported ethnicity and pregnancy outcome. PCoA groups are shown in relation to ethnicity in (A) early (10-15^{+6} weeks gestation) and (B) late (16-24^{+6} weeks gestation) samples. (C) Distribution of PCoA groups based on ethnicity and term outcome in early samples compared to late samples (D). sPTB37: spontaneous preterm birth <37 weeks' gestation. Early samples comprise of: Black N=67 (16 sPTB37); White N=207 (23 sPTB37); Other N=31 (11 sPTB37). Late samples comprise of: Black N=65 (13 sPTB37); White N=218 (28 sPTB37); Other N=30 (9 sPTB37)

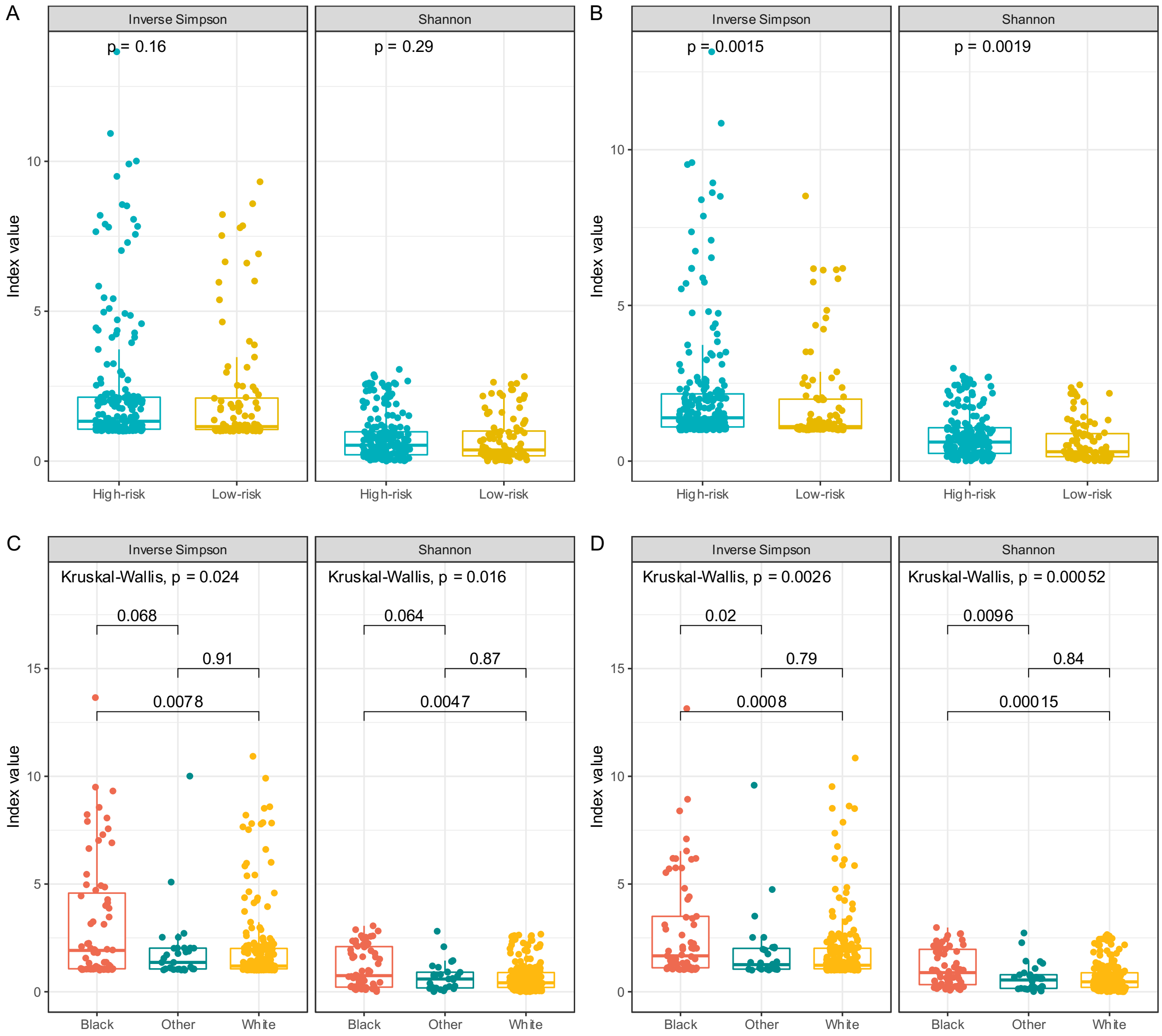

Figure S9: Alpha diversity analyses at OTU level of species richness within cervicovaginal fluid. Alpha diversity significance based on Wilcoxon and Kruskal-Wallis calculated on Inverse Simpson and Shannon indexes. (A, C) Early cervicovaginal fluid samples (10-15^{+6}weeks gestation); (B, D) late samples (16-24^{+6} weeks gestation). Analyses based (A-B) on participant pregnancy risk status at study entry, (C-D) on ethnicity. Horizontal line and boxes represent median and interquartile range.

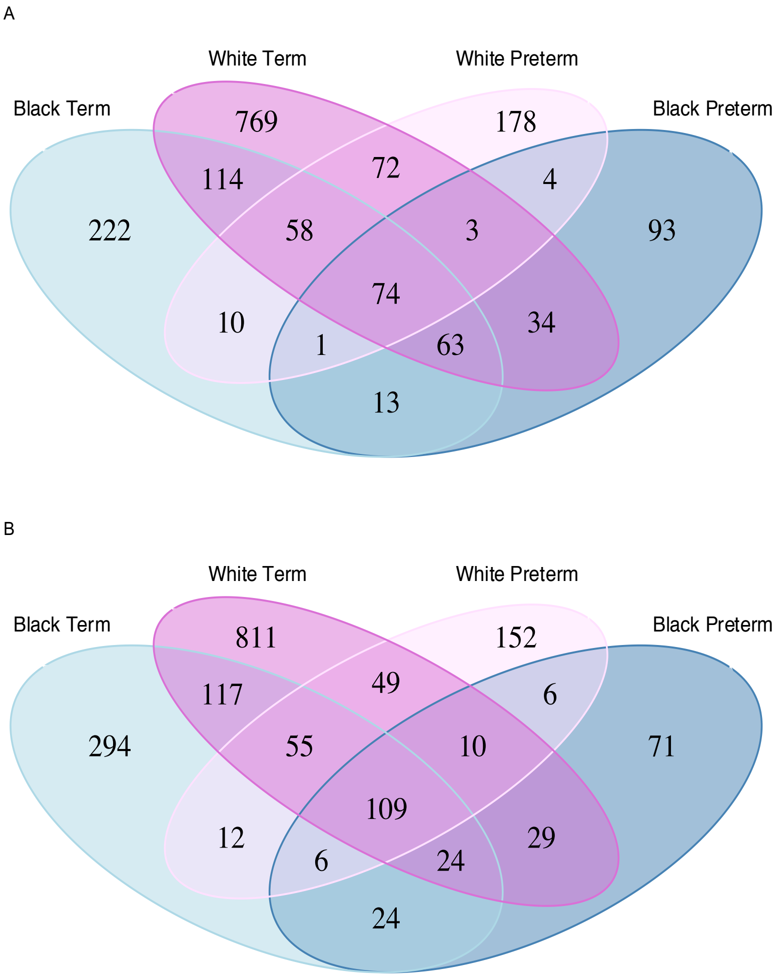

Figure S10: Venn Diagram of cervicovaginal fluid OTUs composition. Comparison of OTUs assignments for White and Black women delivering at term or sPTB37 (spontaneous preterm birth > 37 weeks' gestation) in (A) early cervicovaginal fluid samples (10-15^{+6} weeks gestation) and (B) late samples (16-23^{+6} weeks gestation).

A

B

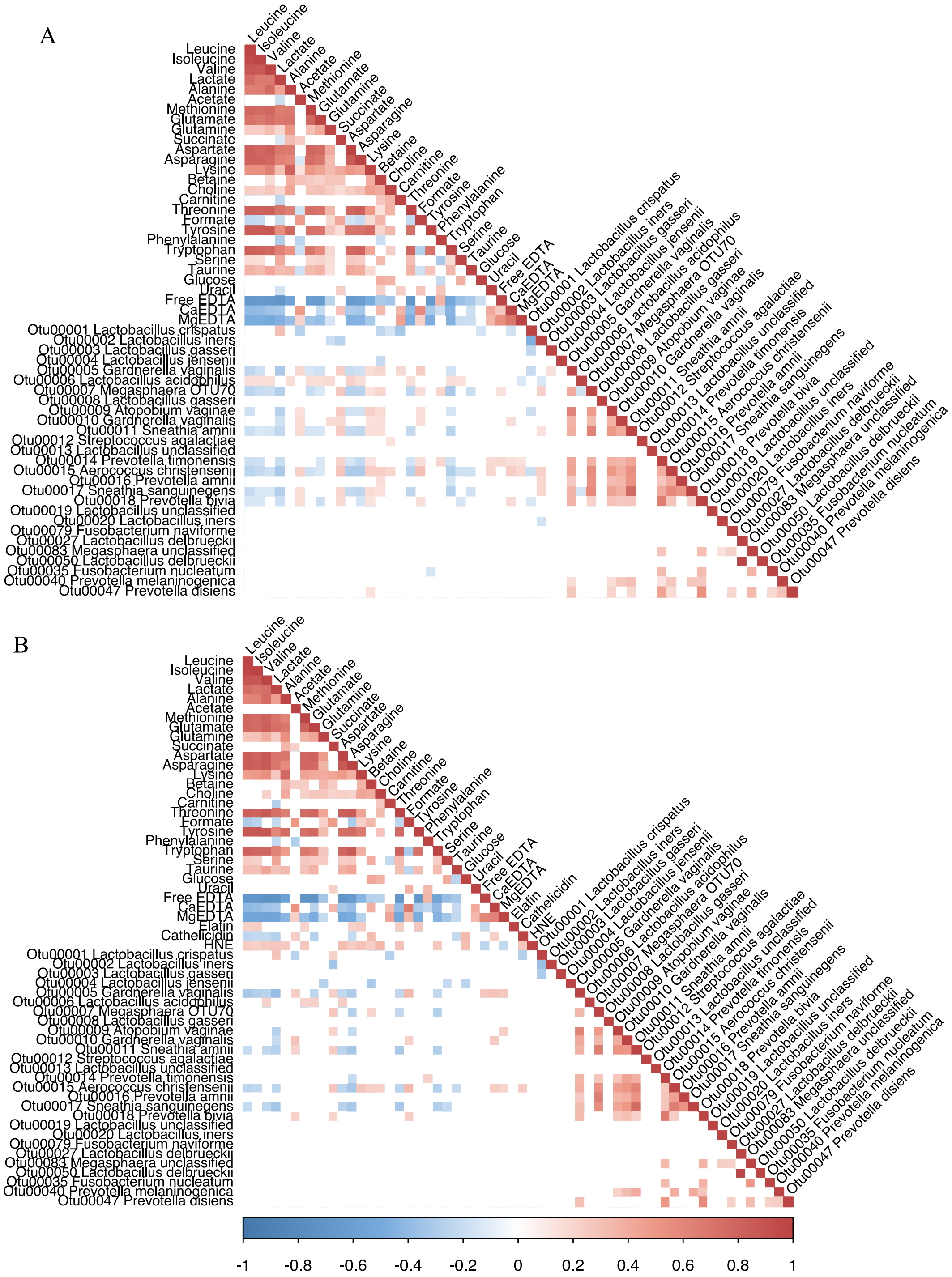

Figure S11: Spearman Correlation analyses of cervicovaginal fluid OTUs, metabolites and host response proteins in late samples (16-23^{+6} weeks gestation). (A) OTUs and metabolites (N= 305); (B) OTUs, metabolites, elafin, cathelicidin and HNE (N=154). OTUs are selected as most abundant i.e. filtered out OTUs with less than 1% average abundance and identified via LEfSe analyses for spontaneous preterm birth (sPTB <37 weeks). Only correlations (blue (negative) and red (positive)) with adjusted p values <0.05 are shown

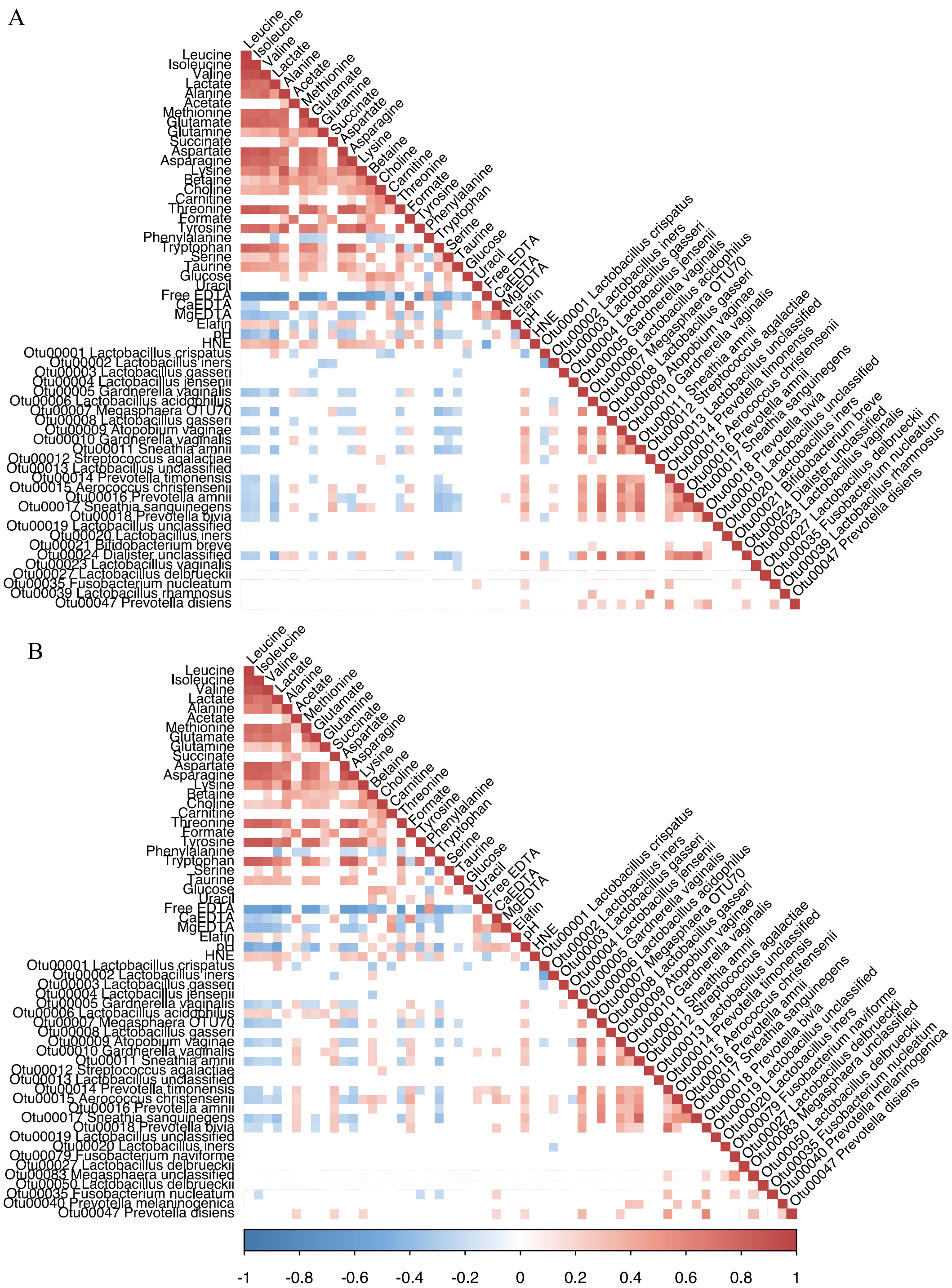

Figure S12: Spearman Correlation analyses of cervicovaginal fluid from whole cohort to include pH. (A) OTUs, metabolites, human neutrophil elastase (HNE), pH and elafin (N=164) in early samples, and in late samples (B) (N=164). OTUs selected as follows: i) showing more than 1% average abundance ii) identified via LEfSe analyses as associated to spontaneous preterm birth (sPTB <37 weeks). Only correlation (blue (negative) and red (positive)) with adjusted p values <0.05 are shown.

Table S1: Participant demographics. Characteristics of participants providing samples in at least one time point (either early or late gestation) for microbiome, metabolite and host defense protein/peptide analysis. IUD pregnancies were removed; sPTB includes preterm prelabor rupture of membranes (PPROM, n=29) and mid-trimester loss (n=11). sPTB37: spontaneous preterm birth <37 weeks' gestation; sPTB34: spontaneous preterm birth <34 weeks' gestation.

**Characteristic**	**Subcategory**	**Term**	**sPTB37(sPTB34)**	**Total Women**
Count N (%)		286 (82.7%)	60 (27 sPTB34) (17.3%)	346
Maternal age (Years ± SD) at booking		32.4±4.8	33.6±6.2	32.6±5.1
Body Mass Index kg/m^2) at booking		25.5±5.5 (N=285)	27.0±4.9 (N=60)	25.7±5.5 (N=345)
	White	24.43±4.53 (N=204)	24.87±3.96 (N=31)	24.49±4.45 (N=235)
	Black	29.33±6.91 (N= 57)	29.57±5.01 (N=18)	29.38±6.49 (N= 75)
	Other (Asian and unknown)	25.77±6.66 (N=24)	27.84±3.97 (N=11)	26.42±5.97 (N= 35)
Ethnicity N (%)	White	205 (86.9%)	31 (11 sPTB34) (13.1%)	236 (100%)
	Black	57 (76.0%)	18 (10 sPTB34) (24.0%)	75 (100%)
	Other (Asian and unknown)	24 (68.6%)	11 (6 sPTB34) (31.4%)	35 (100%)
Risk status N (%)	Low risk	102 (96.23%)	4 (0 sPTB34) (3.77%)	106
	Low risk White	80 (100%)	0 (0%)	80 (100%)
	Low risk Black	13 (81.2%)	3 (18.8%)	16 (100%)
	Low risk other	9 (90%)	1 (10%)	10 (100%)
	High risk	184 (76.67%)	56 (27 sPTB34) (23.33%)	240
	High risk White	125 (80.1%)	31 (19.9%)	156 (100%)
	High risk Black	44 (74.6%)	15 (25.4%)	59 (100%)
	High risk other	15 (60%)	10 (40%)	25 (100%)

Smoking status N	Current smoker	17	2	19
	White	14	1	15
	Black	2	0	2
	Other	1	1	2
	Ex-smoker (gave up in current pregnancy)	17	3	20
	White	13	3	16
	Black	4	1	5
	Other	0	0	0
	Ex (gave up before current pregnancy	40	9	49
	White	31	8	39
	Black	5	0	5
	Other	0	0	0
	Never smoked	211	44	255
	White	146	19	
	Black	46	16	
	Other	19	9	
	Missing data	1	2	3
Short cervix <25 mm before 24 weeks gestation (N)	High risk	25	24 (18 sPTB34)	49
High-risk with short cervix <25 mm before 24 weeks gestation	Black	16	10 (8 sPTB34)	26
	White	9	9 (6 sPTB34)	18
	Other	5	5 (4 sPTB34)	10

Table S2: **Exploration of cervicovaginal fluid (CVF) components.** Comparison of principal component analyses (PCoA) groups in the early samples (10-15^{+6} weeks gestation) with cervicovaginal fluid metabolites (whole cohort) and host defense molecules. Significant differences (Wilcoxon p-value <0.05) indicated by green (lower) or orange (higher) in A or B versus other PCoA groups.

PCOA group	A v B	A v C	A v D	A v E	B v C	B v D	C v D
**CVF metabolites**							
Acetate							
Alanine							
Asparagine							
Aspartate							
Betaine							
Ca EDTA							
Carnitine							
Choline							
Formate							
Glucose							
Glutamate							
Glutamine							
Isoleucine							
Lactate							
Leucine							
Lysine							
Methionine							
Mg EDTA							
Phenylalanine							
Serine							
Succinate							
Taurine							
Threonine							
Tryptophan							
Tyrosine							
Uracil							
Valine							
**Other CVF measurements**							
pH							
Elafin							
Cathelicidin							
HNE							

Number of samples per PCoA group for comparisons between i. metabolites: A = 89, B = 31, C = 115, D = 64, E = 6; ii. pH: A = 55, B=19, C=62, D=39, E=4; iii. Elafin: A= 85, B=29, C=111, D=61, E=6; iv. cathelicidin measurements: A= 41, B=16, C=76, D=37, E=4; and v. human neutrophil elastase (HNE): A= 84, B=29, C=107, D=57, E=6. One further metabolite was detected in CVF but removed from our analyses as propylene glycol was a contaminant from the lubricant used for cervical length measurement in high risk women.

Table S3: Orthogonal Projections to Latent Structures – Discriminant Analysis (OPLS-DA) of cervicovaginal fluid metabolites differences. R^2X and R^2Y: explained variation, goodness of fit for X/OTU. Q^2X: goodness of prediction, explain the predicted variation. RAMSEE: Root Mean Squared Error of Estimation. pR^2Y and pQ^2: permuted R^2Y and Q^2Y; these values provide a baseline for Q2 above which a model can discriminate according to the indicated binary classification.

**Comparison**	**R^2X (cum)**	**R^2Y (cum)**	**Q^2 (cum)**	**RMSEE**	**pR^2Y**	**pQ^2**
Ethnicity: White women and Black women early samples	0.445	0.174	0.112	0.394	0.05	0.05
Ethnicity: White women and Black women late samples	0.468	0.168	0.123	0.391	0.05	0.05
Risk: Low-risk and High-risk women in late samples (White and Black women only)	0.396	0.134	0.044	0.437	0.05	0.05

Table S4: LDA (Linear Discriminant Analysis) Effect Size (LEfSe) of cervicovaginal fluid OTUs in relation to term and preterm (<37 weeks) birth. Analyses for cervicovaginal fluid samples collected during early (10-15^{+6} weeks) and late (16-23^{+6} weeks) pregnancy. Associations between OTUs and term delivery or sPTB <37 weeks (sPTB37) in the whole cohort and stratified by ethnicity assessed by LEfSe. All data have an LDA score > 3 and p-value < 0.05. Table show OTUs with relative abundance overall >0.5% across all samples, in bold OTUs >1% abundance.

**Sampling time**	**Direction**	**OTU and species / taxon identification**	**LDA**	**p-value**
All women				
Early Pregnancy	Term	**OTU1 *L. crispatus***	4.959	0.005
		**OTU6 *L. acidophilus***	3.848	0.021
	sPTB37	OTU27 *L. delbrueckii*	3.720	0.001
		**OTU3 *L. gasseri***	4.525	0.021
		**OTU21 *Bifidobacterium breve***	4.024	0.009
		OTU18 *Prevotella bivia*	3.328	0.023
Late Pregnancy	Term	**OTU1 *L. crispatus***	5.032	0.000
	sPTB37	OTU27 *L. delbrueckii*	3.588	0.022
		OTU18 *Prevotella bivia*	3.365	0.001
		OTU35 *Fusobacterium nucleatum*	3.710	0.001
		OTU47 *Prevotella disiens*	3.099	0.014
White women				
Early Pregnancy	Term	**OTU1 *L. crispatus***	4.982	0.006
	sPTB37	OTU27 *L. delbrueckii*	3.973	0.005
		**OTU3 *L. gasseri***	4.818	0.014
Late Pregnancy	Term	**OTU1 *L. crispatus***	5.078	0.000
	sPTB37	OTU27 *L. delbrueckii*	4.037	0.009
		OTU18 *Prevotella bivia*	3.452	0.030
		OTU39 *L. rhamnosus*	3.228	0.030
		OTU26 *Dialister micraerophilus*	3.221	0.001
		OTU47 *Prevotella disiens*	3.159	0.036
Black women				
Early Pregnancy	sPTB37	OTU23 *L. vaginalis*	3.032	0.025
		**OTU21 *Bifidobacterium breve***	4.398	0.010
		**OTU6 *L. acidophilus***	3.274	0.004
Late Pregnancy	Term	**OTU3 *L. gasseri***	4.346	0.050
	sPTB37	OTU35 *Fusobacterium nucleatum*	3.791	0.023
		OTU18 *Prevotella bivia*	3.522	0.010

Table S5: LDA (Linear Discriminant Analysis) Effect Size (LEfSe) of cervicovaginal fluid OTUs in relation to term and preterm (<34 weeks) birth. Analyses for cervicovaginal fluid samples collected during early (10-15^{+6} weeks) and late (16-23^{+6} weeks) pregnancy. LEfSe association at OTUs level in the whole cohort. Cervicovaginal OTUs with bacterial taxonomy associated that significantly correlate with term or sPTB34 (spontaneous preterm birth less than 34 weeks' gestation) in all women, White or Black women only. All data have an LDA score >3 and p-value <0.05. Table shows OTUs with relative abundance overall >0.5% across all samples, in bold OTUs >1% abundance.

**Sampling time**	**Direction**	**OTU and species / taxon identification**	**LDA**	**p-value**
All women				
Early Pregnancy	Term	**OTU7 *Megasphaera* "OTU70"**	4.103	0.029
		**OTU6 *Lactobacillus acidophilus***	3.914	0.044
		**OTU16 *Prevotella amnii***	3.474	0.042
	sPTB34	**OTU3 *Lactobacillus gasseri***	4.736	0.024
		**OTU21 *Bifidobacterium breve***	4.323	0.043
		OTU27 *Lactobacillus delbrueckii*	4.003	7.05E-07
Late Pregnancy	Term	**OTU1 *Lactobacillus crispatus***	5.06	0.025
	sPTB34	**OTU14 *Prevotella timonensis***	4.1	0.006
		OTU27 *Lactobacillus delbrueckii*	4.064	1.92E-04
		OTU35 *Fusobacterium nucleatum*	4.042	2.45E-07
		OTU18 *Prevotella bivia*	3.768	4.63E-06
		OTU26 *Dialister micraerophilus*	3.291	1.70E-05
		OTU24 *Dialister* unclassified	3.116	0.022
White women				
Early Pregnancy	sPTB34	**OTU3 *Lactobacillus gasseri***	5.05	0.0162
		OTU27 *Lactobacillus delbrueckii*	4.579	6.12E-07
		OTU39 *Lactobacillus rhamnosus*	3.101	0.0005
Late Pregnancy	Term	**OTU1 *Lactobacillus crispatus***	5.205	0.0315
	sPTB34	OTU27 *Lactobacillus delbrueckii*	4.188	5.10E-06
		OTU18 *Prevotella bivia*	3.823	0.0004
		OTU26 *Dialister micraerophilus*	3.436	3.02E-06
		OTU39 *Lactobacillus rhamnosus*	3.215	0.0046
Black women				
Early Pregnancy	Term	**OTU7 *Megasphaera* "OTU70"**	4.382	0.027
	sPTB34	**OTU21 *Bifidobacterium breve***	4.676	0.034
Late Pregnancy	Term	**OTU12 *Streptococcus agalactiae***	4.042	0.011
	sPTB34	OTU35 *Fusobacterium nucleatum*	4.292	2.97E-04
		OTU18 *Prevotella bivia*	3.721	0.032
		OTU45 *Mobiluncus mulieris*	3.33	0.008

Table S6: Composite metabolite prediction model for spontaneous preterm birth <37 weeks. Stepwise logistic regression including ethnicity and cervicovaginal fluid metabolites (detected using NMR) in the model, identified seven metabolites as a useful composite indicator of risk of spontaneous preterm birth <37 weeks (sPTB37). Data from N = 618 CVF samples. Robust standard errors and hence confidence interval and p-values, were adjusted for up to two measurements on each participant (n=341). Receiver operator characteristic curves given for overall test; at two different gestational sampling points and stratified for self-reported ethnicity. Odds ratios given for the contribution of individual metabolites to the overall prediction. For the seven metabolites, the Odds Ratios show the impact of a 1 standard deviation change in metabolite levels on chance of SPTB (standard deviations for leucine 0.538, tyrosine 0.202, lactate 5.62, betaine 0.209, acetate 1.88, Ca^{2+} 2.26, aspartate 0.0279, glucose, 0.423).

Composite metabolite prediction model	ROC Area<br>(Confidence interval)
sPTB37	0.752 (0.699 to 0.806)
Test at 10-15^{+6} weeks	0.748 (0.671 to 0.824)
Test at 16-23^{+6} weeks	0.763 (0.689 to 0.836)
Composite metabolite model by ethnicity	
Test (Black women)	0.716 (0.607 to 0.826)
Test (White women)	0.750 (0.677 to 0.822)
Test (Other)	0.751 (0.622 to 0.881)
Individual metabolites in composite	Odds Ratios (Confidence Interval)
Leucine	3.118 (1.616 to 6.017)
Tyrosine	0.023 (0.002 to 0.251)
Aspartate	1.675 (1.035 to 2.712)
Lactate	0.432 (0.277 to 0.675)
Betaine	1.365 (1.029 to 1.812)
Acetate	1.610 (1.220 to 2.124)
Ca^{2+}	1.378 (1.003 to 1.894)

Table S7: Logistic regression of significant cervicovaginal three metabolites (standardized) to predict spontaneous preterm birth <34 weeks (sPTB34). N=618 samples from 341 women. Robust standard errors and hence confidence interval and p-values, were adjusted for up to two measurements per participant. For Ca^{2+}, aspartate and glucose, Odds Ratios show the impact of a 1 standard deviation change in metabolite levels on chance of sPTB34 (standard deviations for Ca^{2+} 2.26; aspartate 0.0279; glucose, 0.423).

Composite prediction model	ROC Area	Confidence Interval
sPTB34	0.701	0.626 to 0.776
Individual metabolites (n=341; n=618)	Odds Ratio	Confidence Interval
Glucose	1.269	1.129 to 1.426
Ca^{2+}	1.86291	1.298 to 2.673
Aspartate	1.767967	1.295 to 2.414
In order rejected from the model at P<0.05		
Free EDTA, methionine, betaine, acetate		

Table S8: Stepwise logistic regression model of phylotypes. Stepwise logistic regression including ethnicity and the 9 most abundant cervicovaginal fluid OTUs (detected using 16s rRNA sequencing) in the model, identified Black and Other ethnicities and the OTUs associated with L. acidophilus and L. crispatus as being significantly associated with risk of spontaneous preterm birth <34 weeks. Data from N = 618 CVF samples. Robust standard errors (not shown), and hence confidence interval and p-values, were adjusted for up to two measurements on each participant (n=341). Odds Ratio of the bacteria show the impact of a 1% change in the prevalence on chance of preterm birth

	Odds Ratio	Confidence interval
L. acidophilus	0.008	0.001 to 0.115
L. crispatus	0.233	0.059 to 0.921
Black ethnicity	2.489	0.915 to 6.77
Other ethnicity	4.374	1.402 to 13.642
In order, rejected from model at P>0.05		
S. amnii, A. vaginae, G. vaginalis, L. gasseri, L. jensenii, L. iners, M. OTU70		

Table S9: Logistic regression metabolites and phylotypes for spontaneous preterm birth <34 weeks (sPTB34). Logistic regression of significant cervicovaginal fluid metabolites (standardized), *L. crispatus* and *L. acidophilus* phylotypes proportions to predict sPTB34. N=618 samples from 341 women. Robust standard errors and hence confidence interval and p-values, were adjusted for up to two measurements per participant. For Ca2+, aspartate and glucose, Odds Ratios show the impact of a 1 standard deviation change in metabolite levels on chance of sPTB (standard deviations for Ca^{2+} 2.26; aspartate 0.0279; glucose, 0.423). For *L. crispatus* and *L. acidophilus* Odds Ratios show the impact of a 1% change in prevalence.

Composite prediction model	ROC Area	Confidence Interval
sPTB34	0.758	0.692 to 0.823
Test at $10\text{-}15^{+6}$ weeks	0.755	0.655 to 0.855
Test at $16\text{-}23^{+6}$ weeks	0.758	0.676 to 0.840
Composite metabolite prediction by ethnicity		
Test (black women)	0.594	0.452 to 0.735
Test (white women)	0.835	0.765 to 0.906
Test (Other)	0.716	0.541 to 0.891
Individual metabolites in composite (n=341; n=618)	Odds Ratios	Confidence Interval
L. crispatus	0.986	0.973 to 0.999
Ca^{2+}	1.712	1.150 to 1.548
Aspartate	1.883	1.314 to 2.697
L. acidophilus	0.943	0.921 to 0.967
Glucose	1.230	1.085 to 1.395

Table S10: Characterization of the 16S microbiome based on species-level composition by samples. Sequences were assigned to the vaginal database taxonomy by means of the Wang approach.
